# Characterization of Toxin Complex Gene Clusters and Insect Toxicity of Bacteria Representing Four Subgroups of *Pseudomonas fluorescens*

**DOI:** 10.1371/journal.pone.0161120

**Published:** 2016-08-31

**Authors:** Lorena I. Rangel, Marcella D. Henkels, Brenda T. Shaffer, Francesca L. Walker, Edward W. Davis, Virginia O. Stockwell, Denny Bruck, Barbara J. Taylor, Joyce E. Loper

**Affiliations:** 1 Department of Botany and Plant Pathology, Oregon State University, Corvallis, Oregon, United States of America; 2 Agricultural Research Service, US Department of Agriculture, Horticultural Crops Research Laboratory, Corvallis, Oregon, United States of America; 3 Department of Integrative Biology, Oregon State University, Corvallis, Oregon, United States of America; International Centre for Genetic Engineering and Biotechnology, ITALY

## Abstract

Ten strains representing four lineages of the *Pseudomonas fluorescens* group (*P*. *chlororaphis*, *P*. *corrugata*, *P*. *koreensis*, and *P*. *fluorescens* subgroups) were evaluated for toxicity to the tobacco hornworm *Manduca sexta* and the common fruit fly *Drosophila melanogaster*. The three strains within the *P*. *chlororaphis* subgroup exhibited both oral and injectable toxicity to the lepidopteran *M*. *sexta*. All three strains have the gene cluster encoding the FitD insect toxin and a Δ*fitD* mutant of *P*. *protegens* strain Pf-5 exhibited diminished oral toxicity compared to the wildtype strain. Only one of the three strains, *P*. *protegens* Pf-5, exhibited substantial levels of oral toxicity against the dipteran *D*. *melanogaster*. Three strains in the *P*. *fluorescens* subgroup, which lack *fitD*, consistently showed significant levels of injectable toxicity against *M*. *sexta*. In contrast, the oral toxicity of these strains against *D*. *melanogaster* was variable between experiments, with only one strain, *Pseudomonas* sp. BG33R, causing significant levels of mortality in repeated experiments. Toxin complex (Tc) gene clusters, which encode insecticidal properties in *Photorhabdus luminescens*, were identified in the genomes of seven of the ten strains evaluated in this study. Within those seven genomes, six types of Tc gene clusters were identified, distinguished by gene content, organization and genomic location, but no correlation was observed between the presence of Tc genes and insect toxicity of the evaluated strains. Our results demonstrate that members of the *P*. *fluorescens* group have the capacity to kill insects by both FitD-dependent and independent mechanisms.

## Introduction

*Pseudomonas* is a diverse genus of γ-Proteobacteria known for its ubiquity in the natural world, capacity to utilize a striking variety of organic compounds as energy sources, and production of a remarkable array of exoenzymes, toxins, and secondary metabolites. The genus currently comprises at least 144 species [1] exhibiting varied lifestyles in a wide range of environments, including soil, water, plant surfaces and animals. Within the genus, the *Pseudomonas fluorescens* group is particularly heterogeneous, encompassing bacteria that have been classified into many subgroups and more than 60 named species, including *P*. *protegens*, *P*. *chlororaphis*, *P*. *brassicacearum*, and *P*. *fluorescens* itself [2]. Many plant-associated bacteria in these species have the capacity to suppress diseases caused by a spectrum of bacterial, fungal, and oomycete pathogens [3]. These beneficial bacteria are important components of the soil and plant microbiome contributing to plant health [4,5] and some strains have been used commercially for biological control of plant disease [6,7]. Certain strains within the *P*. *fluorescens* group exhibit insecticidal activity [8,9,10,11,12,13,14], opening the potential to use these bacteria for management of diseases and insect pests of plants.

**Fig 1:**
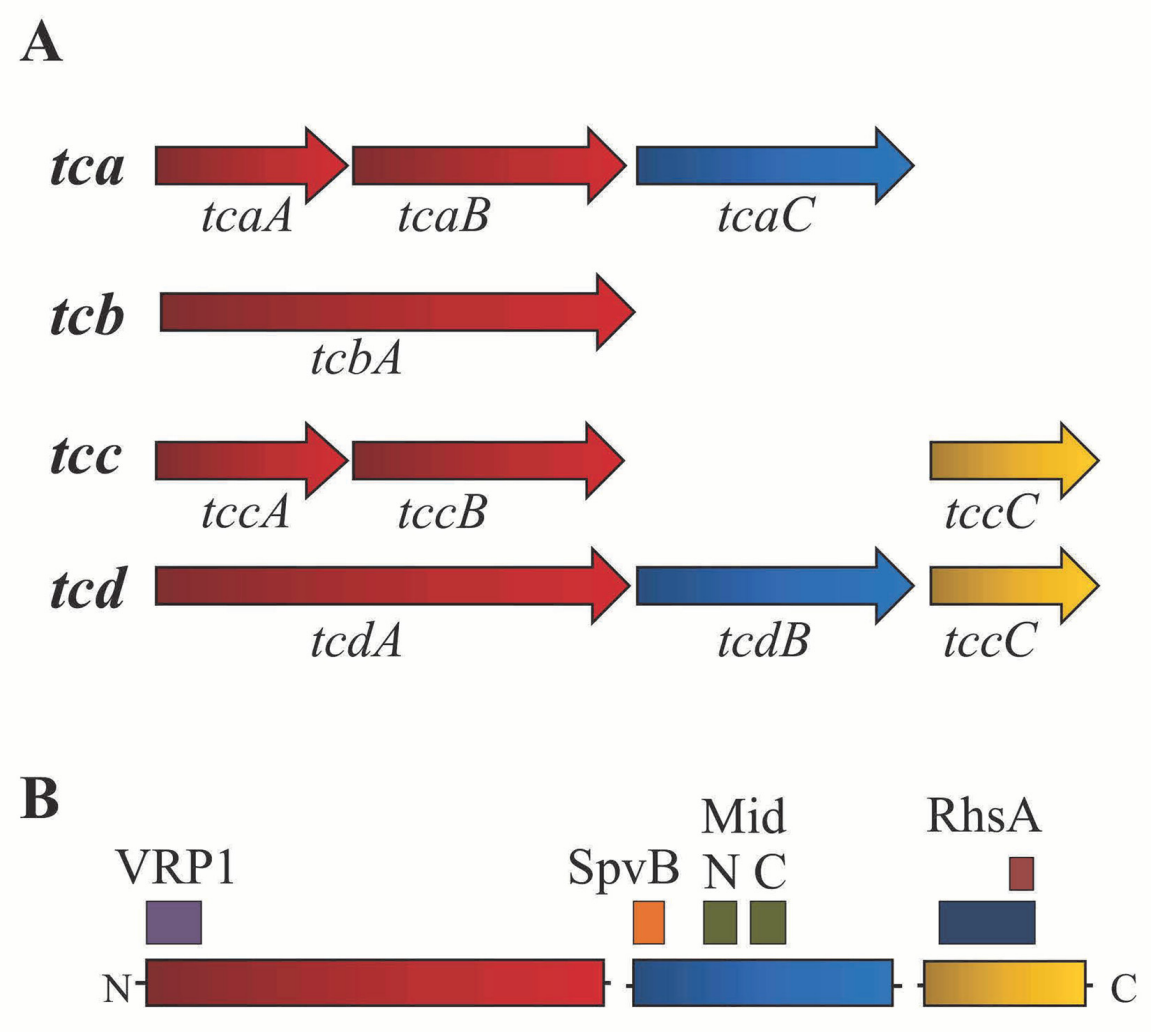
Tc gene clusters in *Photorhabdus luminescens* W14. (A) The four Tc gene clusters (*tca*, *tcb*, *tcc* and *tcd*) present in the genome of *P*. *luminescens* W14. Genes are colored according to the Tc component encoded: red (Component A), blue (Component B) and yellow (Component C). Component A can be encoded by two genes or by a single, large gene. (B) Conserved domains present in each Tc component (A, B and C). The A component (TcaA/TcdA) has a conserved VRP1 domain near the N-terminus. The B component (TcaC/TcdB) has the SpvB domain near the N-terminus and the MidN and MidC domains near the center. The C component (TccC) has an RhsA domain with an Rhs repeat-associated core domain.

Two types of insect toxin genes have been identified in strains of the *P*. *fluorescens* group and both types are similar to genes first described in entomopathogenic bacteria in the genera *Xenorhabdus* and *Photorhabdus*. A gene encoding FitD (*f**luorescens* insecticidal toxin) is present in the genomes of *P*. *protegens* [11] and the related species *P*. *chlororaphis* [15,16]. FitD is similar to the Mcf (“Makes caterpillars floppy”) toxin of *Photorhabdus luminescens*, which exhibits injectable toxicity towards insects via apoptosis [17,18,19]. When injected into larvae of the tobacco hornworm *Manduca sexta* or the wax moth *Galleria mellonella*, *fitD*-containing strains of *P*. *protegens* are lethal [11]. Furthermore, FitD is a major determinant of the oral toxicity of *P*. *protegens* CHA0 and *P*. *chlororaphis* PCL1391 to three agriculturally-important lepidopteran insect pests, the African cotton leafworm *Spodoptera littoralis*, the tobacco budworm *Heliothis virescens*, and the diamondback moth *Plutella xylostella* [13]. In addition to *fitD*, some strains in the *P*. *fluorescens* group [16,20] have genes similar to those encoding Toxin complex (Tc) proteins, which are best characterized in the entomopathogen *Photorhabdus luminescens* strain W14 [21]. Tc toxins have three components (A, B, and C), each of which have conserved domains (Fig 1) [20]. All three components are necessary for full toxicity [22] with the A component serving as the primary toxin and the B and C components thought to be potentiators enhancing toxicity of the protein complex [21,23]. Tc genes are present in the genomes of many bacteria, including species without known associations with insects. Within the genus *Pseudomonas*, Tc gene clusters have been described in *P*. *syringae* pv. *syringae* B728a and *P*. *fluorescens* Pf0-1 [20] and a *tccC* gene has been associated with insect toxicity of *Pseudomonas taiwanensis* [14].

**Fig 2:**
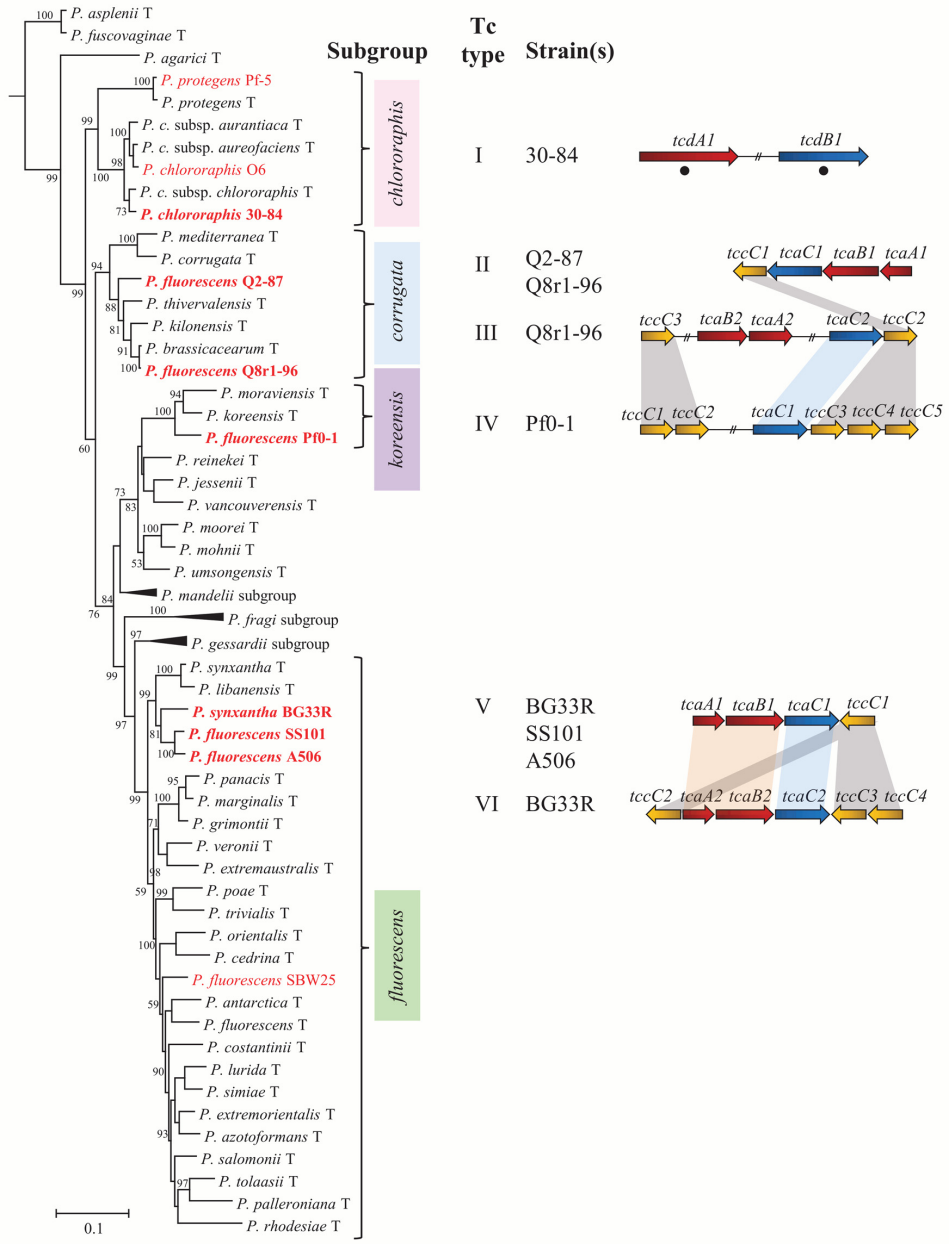
Seven of the ten strains of *Pseudomonas* spp. evaluated in this study have Tc clusters. The ten strains evaluated in this study fall into four subgroups within the *P*. *fluorescens* group, as shown in a phylogenetic tree based on concatenated alignments of *gyrB*, *rpoB*, *rpoD* and 16S rRNA of the type strains within the *P*. *fluorescens* group. The ten strains examined in this study are shown in red font and subgroups containing these strains are labelled to the right (pink, *chlororaphis* subgroup; blue, *corrugata* subgroup; purple, *koreensis* subgroup; green, *fluorescens* subgroup). The tree is artificially rooted on the type strain of *P*. *aeruginosa*. Subgroups lacking any of the ten strains are collapsed and labeled. Bootstrap support less than 50% is not shown. Branch lengths indicate the number of nucleotide substitutions per site. Strains evaluated in this study that contain a Tc cluster are shown in bold, red font and are also listed to the right of the tree. Tc cluster Types I-VI are distinguished from one another by gene organization and genome location. Genes are colored according to the Tc component encoded: red (component A), blue (component B) and yellow (component C). Type I: 30–84 (Pchl3084_2947 and Pchl3084_2950); Type II: Q2-87 (PflQ2_0667–0670) and Q8r1-96 (PflQ8_0736–0739); Type III: Q8r1-96 (PflQ8_4696, PflQ8_4570–4571 and PflQ8_4580–4581); Type IV: Pf0-1 (Pfl01_0947–0948 and Pfl01_4453–4456); Type V: A506 (PflA506_3065–3068), SS101 (PflSS101_2971–2974) and BG33R (PseBG33_3189–3192); Type VI: BG33R (PseBG33_3799–3804). Numbers following gene names distinguish genes within a single genome that encode the same Tc component. Black circles denote genes located on genomic islands. Among genomes, homologous genes, defined by genomic location and phylogenetic relationships, are connected with shading of the same color.

The aims of this study were to evaluate ten strains of the *P*. *fluorescens* group for insect toxicity and to characterize the putative Tc genes (*tcaA*, *tcaB*, *tcaC*, *tcdA*, *tcdB*, *tccC*) in the genomes of these ten strains. The strains tested were isolated from soil, root or leaf surfaces and known for their biological control of plant diseases [16,24,25]. They represent four subgroups (*P*. *chlororaphis*, *P*. *corrugata*, *P*. *koreensis*, and *P*. *fluorescens*) of the *P*. *fluorescens* group (Fig 2). This study demonstrated that the *P*. *fluorescens* group includes strains that are toxic to insects representing two orders (Diptera and Lepidoptera) and that the known insect toxin FitD is responsible for some, but not all, of the insect toxicity of these bacteria.

## Results

### Phylogenetic analysis of ten strains of *Pseudomonas* spp. evaluated for insect toxicity

The ten strains evaluated in this study, which were the subjects of an earlier comparative genomic analysis [16], were placed in four subgroups of *P*. *fluorescens* based on multilocus sequence analysis (MLSA; Fig 2). Previously, the ten strains evaluated in this study were assigned to three sub-clades of the *P*. *fluorescens* group based on an MLSA of strains of *Pseudomonas* spp. with fully-sequenced genomes [16]. By including the type species within the *P*. *fluorescens* group in the MLSA shown in Fig 2, the ten strains were placed in four established subgroups of the *P*. *fluorescens* group. The three strains previously assigned to sub-clade 3 (*P*. *chlororaphis* 30–84, *P*. *chlororaphis* O6, and *P*. *protegens* Pf-5), are in the *P*. *chlororaphis* subgroup. Two strains previously assigned to sub-clade 2, *P*. *brassicacearum* Q8r1-96 and *Pseudomonas* sp. Q2-87, are in the *P*. *corrugata* subgroup. *Pseudomonas* sp. Pf0-1, which was assigned previously to sub-clade 2 [16], is in the *P*. *koreensis* subgroup. The four strains previously assigned to sub-clade 3 (SBW25, A506, SS101, and BG33R) are in the *P*. *fluorescens* subgroup (Fig 2). The results of our current MLSA, which was based on concatenated sequences of the housekeeping genes *gyrB*, *rpoD*, *rpoB*, and 16S rRNA (Fig 2), are congruent with those published recently by other researchers [1,2]. The ten strains of this study were included in the study of Garrido-Sanz et al. [2] and were assigned to the same subgroups in both analyses. Here, the previously defined sub-clades [16] were related to subgroups defined by type species within the *P*. *fluorescens* group.

### Characterization of Tc genes

A bioinformatic survey of the ten strains used in this study identified Tc genes in seven of the ten genomes. A total of 38 Tc genes were identified from a BLASTN search using characterized Tc genes of *P*. *luminescens* W14 as queries. Predicted amino acid sequences for each gene have conserved domains characteristic of the A, B, or C components of Tc proteins present in entomopathogenic bacteria (S1–S3 Tables). Amino acid sequences of candidate *tcaA* genes from five genomes and the much larger *tcdA* of strain 30–84 have a conserved domain called VRP1 (S1 Table). The VRP1 domain corresponds to SpvA, the product of a plasmid-borne gene associated with virulence of *Salmonella* spp. [26]. The VRP1 domain is also present in known TccA, TcaA, TcbA TcdA proteins of entomopathogenic bacteria such as *P*. *luminescens* strain W14 [27]. Amino acid sequences of candidate *tcaC/tcdB* genes from seven genomes also have three conserved domains called SpvB, MidN and MidC (S2 Table). These domains are found in TcaC/TcdB sequences of insect-associated bacteria [27]. The TccC sequences identified in the *Pseudomonas* strains of this study contain the RhsA and Rhs repeat-associated core domains (S3 Table), which are commonly found in secreted bacterial toxins [28]. The presence of these conserved domains in candidate genes provides further evidence for their identity as *tcaA/tcdA*, *tcaC/tcdB* and *tccC* genes.

The organization of Tc genes varies among the seven genomes, falling into six types (I-VI) (Fig 2) distinguished by gene content and organization. Type I, which contains genes encoding putative A and B components, is present in the genome of *P*. *chlororaphis* 30–84. These Tc genes are approximately twice as large as any other Tc gene found in the *P*. *fluorescens* group but are similar in size to *tcdA* and *tcdB of P*. *luminescens* (S1 and S2 Tables) [27]. The Type I Tc gene cluster of strain 30–84 does not include a gene encoding a C component, which is considered a ‘potentiator’ and necessary for full toxicity of the Tc complex of *P*. *luminescens* and *P*. *taiwanensis* [14,21,27]. The Type II Tc cluster, which is present in strains Q8r1-96 and Q2-87, is composed of four contiguous genes (*tcaA1*, *tcaB1*, *tcaC1* and *tccC1*) located at the same site in both genomes (Fig 2). Type III, which is present in strain Q8r1-96 only, is composed of genes for all three Tc components located at three sites distributed over 169 kb of the genome. Although these genes are not contiguous in the genome of Q8r1-96, they could be functional if gene expression is coordinated. The Type IV Tc gene cluster, which is present in strain Pf0-1, lacks an A component but has a single B component and five genes encoding putative C components in two clusters distal to one another in the genome. By analogy to the Tc cluster in *P*. *luminescens* where the A component encodes the primary toxin [21,23], the Type IV cluster may not be functional. Type V, which is present in strains A506, SS101 and BG33R, is composed of four contiguous genes (*tcaA1*, *tcaB1*, *tcaC1* and *tccC1*) located at the same site in all three genomes (Fig 2). Type VI is a single cluster in BG33R containing contiguous genes: *tcaA2*, *tcaB2*, *tcaC2* and three copies of *tccC*. Two strains, Q8r1-96 and BG33R, have two Tc gene clusters of different types.

Phylogenetic analyses were performed using the amino acid sequences of each Tc component present in the seven genomes of the *P*. *fluorescens* group. Trees were created using characterized Tc peptide sequences from *P*. *luminescens* W14 and BLASTP hits for each *P*. *fluorescens* group sequence with greater than 75% query coverage and 50% identity. Phylogenetic relationships support the placement of Tc clusters into the six types described above, with components of the same Tc type from different strains grouping within the same clades (S1–S3 Figs). Genes from different Tc types that fall together within well-supported clades of the phylogenetic trees are shown with shading in Fig 2. The most striking similarities are within the Type V and Type VI clusters, as sequences from these clusters group together in each of the three trees representing the three components of Tc clusters. The peptide sequences of the B and C components of type III and Type IV clusters are also closely related phylogenetically (S2 and S3 Figs) and genes encoding these components are located in identical regions of Q8r1-96 and Pf0-1 genomes. It seems likely that genes encoding the B and C components of these Tc types were inherited from a common ancestor prior to divergence of the clusters, with duplication of genes for the C components in Pf0-1 and either acquisition of the A component genes in strain Q8r1-96 or loss of the A component genes in strain Pf0-1.

Five of the six types of Tc gene clusters (Types II through VI) are in conserved regions of the genome shared by all ten strains of the *P*. *fluorescens* group evaluated in this study. In contrast, the Type I cluster of *P*. *chlororaphis* 30–84 is in a genomic island, unique to *P*. *chlororaphis* 30–84, that has characteristics of a phage, including the presence of genes encoding a phage integrase, a transposase and cointegrate resolution proteins. Sequence bias in this region was found using the Alien Hunter program that searches for regions of genomic plasticity [29]. Furthermore, the G+C content of *tcdA1* (55.4%) and *tcdB1* (56.3%) differ significantly (*P*< 0.001) from the genomic average (62.9%) of strain 30–84, as determined from a chi-square analysis. In our phylogenetic analysis, TcdA1 (Pchl3084_2947) and TcdB1 (Pchl3084_2950) of *P*. *chlororaphis* strain 30–84 are the only peptide sequences to have closely related hits outside of the *Pseudomonas* genus. TcdA1 is closely related to the products of uncharacterized genes in the soil bacterium *Mesorhizobium alhagi* and TcdB1 (Pchl3084_2950) is closely related to a gene product in *Marinomonas posidonica* (S1 and S2 Figs). Based on proximity to genes conferring mobilization functions, gene sequence bias and high identity to gene products of other bacterial taxa, we conclude that the Type I Tc gene cluster was likely acquired through horizontal gene transfer (HGT).

### Strains in the *P*. *fluorescens* group differ in injectable toxicity to *M*. *sexta*

**Fig 3:**
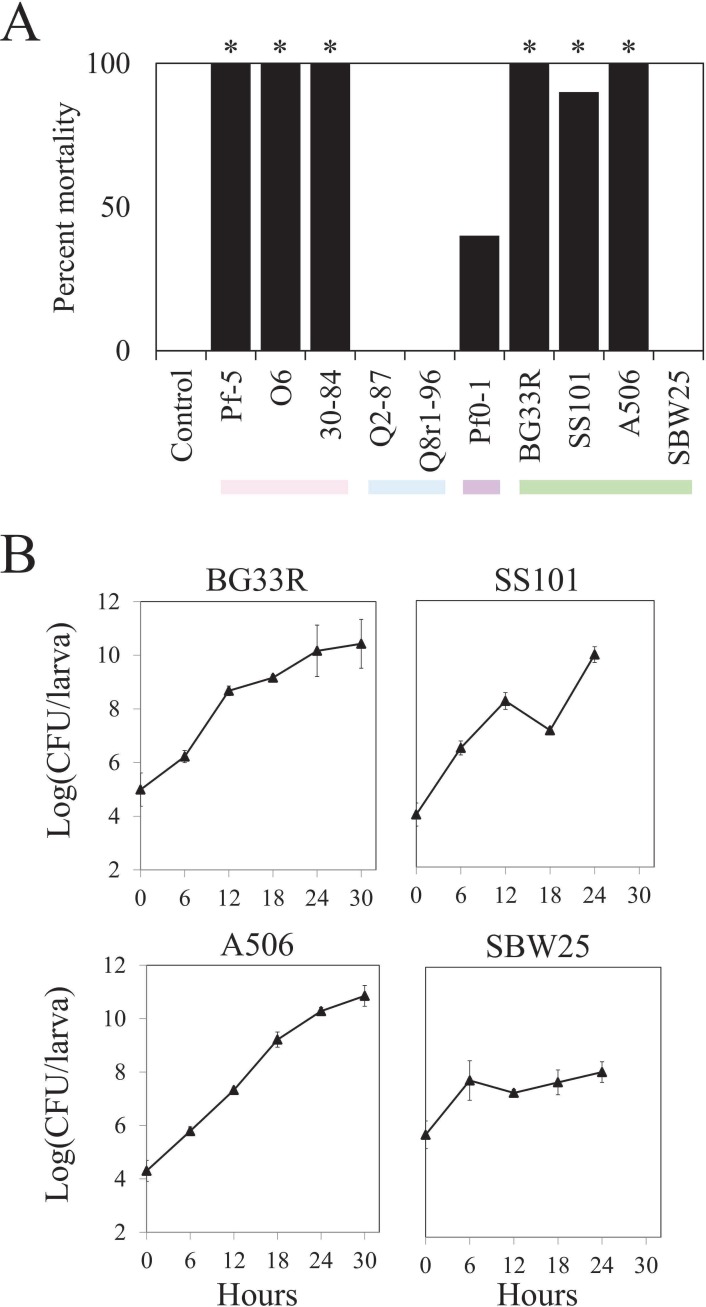
Injectable toxicity of ten strains of *Pseudomonas* spp. and colonization of larvae of *M*. *sexta* by the strains post-injection. (A) Mortality of *M*. *sexta* was assessed 72 h following injection with ca. 5 log (CFU/larva) of the designated strain. Values are from one of three experiments (S4 Fig), each evaluating ten replicate larvae per treatment. Asterisks represent significant differences from the water control (*P*<0.05, d.f. = 1, χ^2^ test). Colors below the graph denote the subgroup of the strain tested for insect toxicity: pink, *P*. *chlororaphis*; blue, *P*. *corrugata*; purple, *P*. *koreensis*; green, *P*. *fluorescens*. (B) Rifampicin-resistant derivatives of strains BG33R, SS101, A506 and SBW25 were injected into ultimate instar larvae of *M*. *sexta* at ca. 5 log (CFU/larva). Internal population sizes of each strain were estimated from ten replicates of surface-sterilized larvae over time. Bacterial population sizes were log transformed and the means and standard errors are shown. No rifampicin-resistant *Pseudomonas* spp. were re-isolated from control larvae injected with sterile water.

Of the ten strains of *P*. *fluorescens* evaluated in this study, six consistently killed *M*. *sexta* when injected at ca. 5 log (CFU/larva) into fifth instar larvae and three strains (Q2-87, Q8r1-96, and SBW25) exhibited no significant toxicity (Fig 3, S4 Fig). One strain, Pf0-1, caused a significant increase in mortality in one experiment (Fig 3), but did not do so in a second experiment (S4 Fig). The greatest levels of hornworm mortality were caused by strains in the *P*. *chlororaphis* subgroup and three of the four strains in the *P*. *fluorescens* subgroup (Fig 3, S4 Fig). At 72 h following injection, the six strains caused similar levels of mortality, but three strains in the *P*. *chlororaphis* subgroup killed the insects within 24 h following injection whereas mortality was slower for insects injected with strains in the *P*. *fluorescens* subgroup (S4 Fig). All three strains (Pf-5, O6 and 30–84) in the *P*. *chlororaphis* subgroup have the *fitD* gene, which encodes the FitD toxin. Because FitD is known to be lethal to *M*. *sexta* and a primary determinant of the injectable insect toxicity phenotype of Pf-5 [11], it is likely that FitD is largely responsible for the injectable toxicity of *P*. *chlororaphis* strains 30–84 and O6 as well. The four strains in the *P*. *fluorescens* subgroup differed in toxicity, with strains A506, BG33R, and SS101 causing significant mortality and strain SBW25 causing no mortality when injected at ca. 5 log (CFU/larva) (Fig 3A). When inoculated at ten-fold higher inoculum densities (ca. 6 log [CFU/larva]), however, all of the strains caused significant mortality in at least one of the two experiments in which they were evaluated (S5 Fig).

The differential lethalities of the four strains in the *P*. *fluorescens* subgroup may be due to their different capacities to colonize the larvae of *M*. *sexta*. To explore this possibility, the growth of each strain in larvae of *M*. *sexta* was assessed over time. Ultimate instar *M*. *sexta* were injected with ca. 5 log (CFU/larva) and sampled every six hours (Fig 3B). The populations of all strains increased over time, and three strains (A506, BG33R, and SS101) reached population sizes of or exceeding 10 log (CFU/larva) at 24 h or 30 h after injection. In contrast to the other strains, SBW25 established lower population sizes in the larvae, reaching only ca. 8 log (CFU/larva) at 24 h, the last time point sampled. SBW25 did not kill larvae of *M*. *sexta* in this experiment whereas the other three strains caused significant levels of mortality (Fig 3A), which suggests a correlation between the toxicity and larval colonization phenotypes of the strains. It is likely that a strain that colonizes larvae would have a greater ability to cause mortality but conversely, it is also possible that a dying animal provides a habitat more conducive to colonization than a healthy animal. The two possibilities were not distinguished in this study.

### A role for FitD in oral toxicity of *P*. *protegens* Pf-5 to *M*. *sexta*

**Fig 4:**
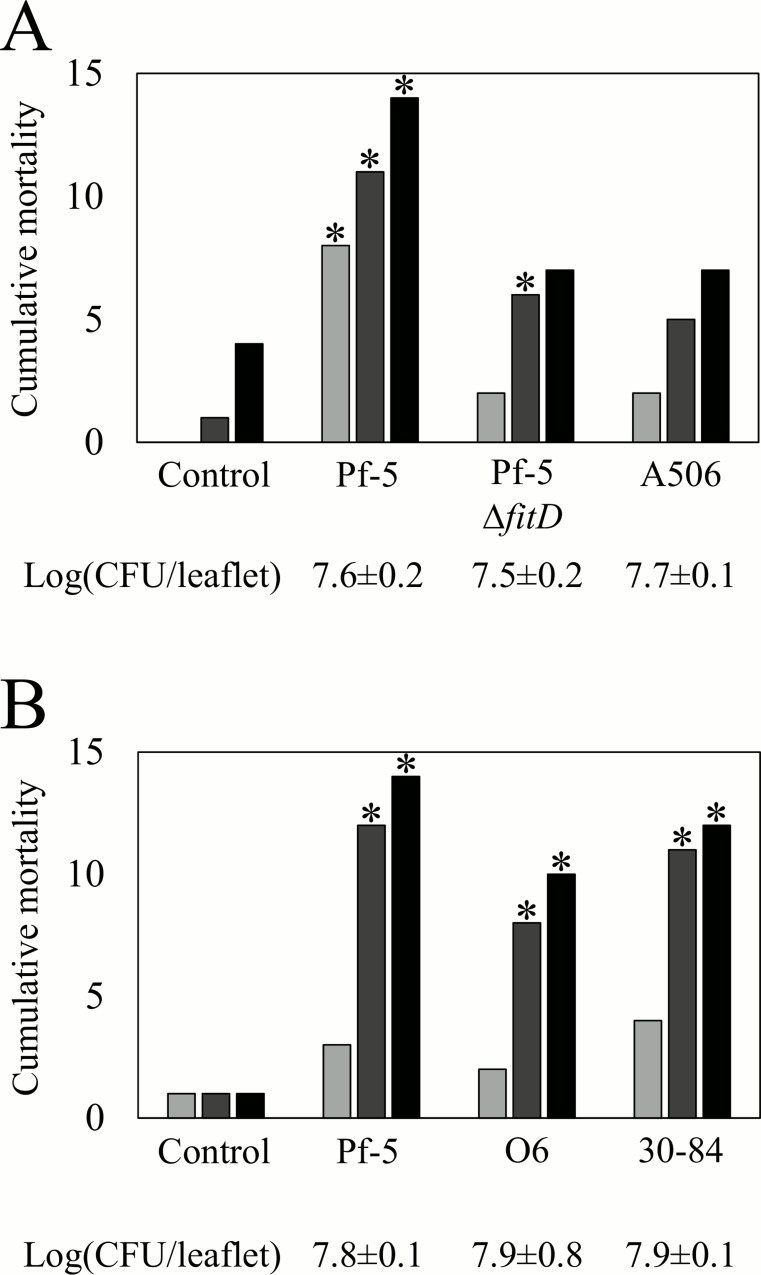
Oral toxicity of the FitD-containing *P*. *chlororaphis* subgroup to *M*. *sexta*. Cumulative mortality of *M*. *sexta* was assessed by counting the number of dead larvae at 2 d (■), 4 d (■) and 6 d (■) after larvae were placed on tomato leaves supporting epiphytic populations of the specified bacterial strain. Bars show the cumulative mortality of larvae on leaves previously inoculated with (A) *P*. *protegens* Pf-5, a Δ*fitD* mutant of Pf-5, and *P*. *fluorescens* A506, a member of the *P*. *fluorescens* subgroup that lacks *fitD*; and (B) three strains in the *P*. *chlororaphis* subgroup, which possess *fitD*. Control larvae were placed on leaves that had not been inoculated with bacteria. Fifteen replicate larvae were evaluated per treatment in each of two experiments that yielded similar results; data presented are from a single experiment. Asterisks represent significant differences from the water control (*P*<0.05, d.f. = 1, χ^2^ test). The epiphytic population size of each strain on tomato leaflets, determined at the time that larvae were placed on the leaves, is shown below each graph.

Due to the established role of FitD in injectable toxicity of Pf-5 to *M*. *sexta* [11], we developed an assay to assess the role of FitD in oral toxicity to the insect. Larvae of *M*. *sexta* were placed on tomato leaves that had been previously inoculated with one of the ten strains of *Pseudomonas* spp. As the larvae fed on the tomato leaves, they ingested cells of the inoculated bacterial strains, which were present at population sizes averaging 7.7 log (CFU/leaflet). Larvae feeding on Pf-5-inoculated leaves had a significantly higher level of mortality than non-inoculated controls in the seven experiments of this study (Fig 4 and S6 Fig). We attribute the mortality caused by Pf-5 to the FitD toxin, as mortality of the larvae on leaves colonized by the Δ*fitD* mutant did not differ significantly (*P*<0.05) from those on non-inoculated leaves (Fig 4A and S6 Fig). Like Pf-5, the two strains of *P*. *chlororaphis* (30–84 and O6), which also have *fitD* [16], caused significant oral toxicity to *M*. *sexta* (Fig 4B and S6 Fig). In contrast, *P*. *fluorescens* A506, a member of the *P*. *fluorescens* subgroup that lacks *fitD*, did not show oral toxicity to *M*. *sexta* (Fig 4A and S6 Fig). Similarly, we tested the other six strains of *Pseudomonas* spp. that lack *fitD*, each in a single experiment, and only BG33R caused significant levels of oral toxicity to *M*. *sexta* (S6 Fig).

### Strains in the *P*. *fluorescens* group differ in oral toxicity to *D*. *melanogaster*

**Fig 5:**
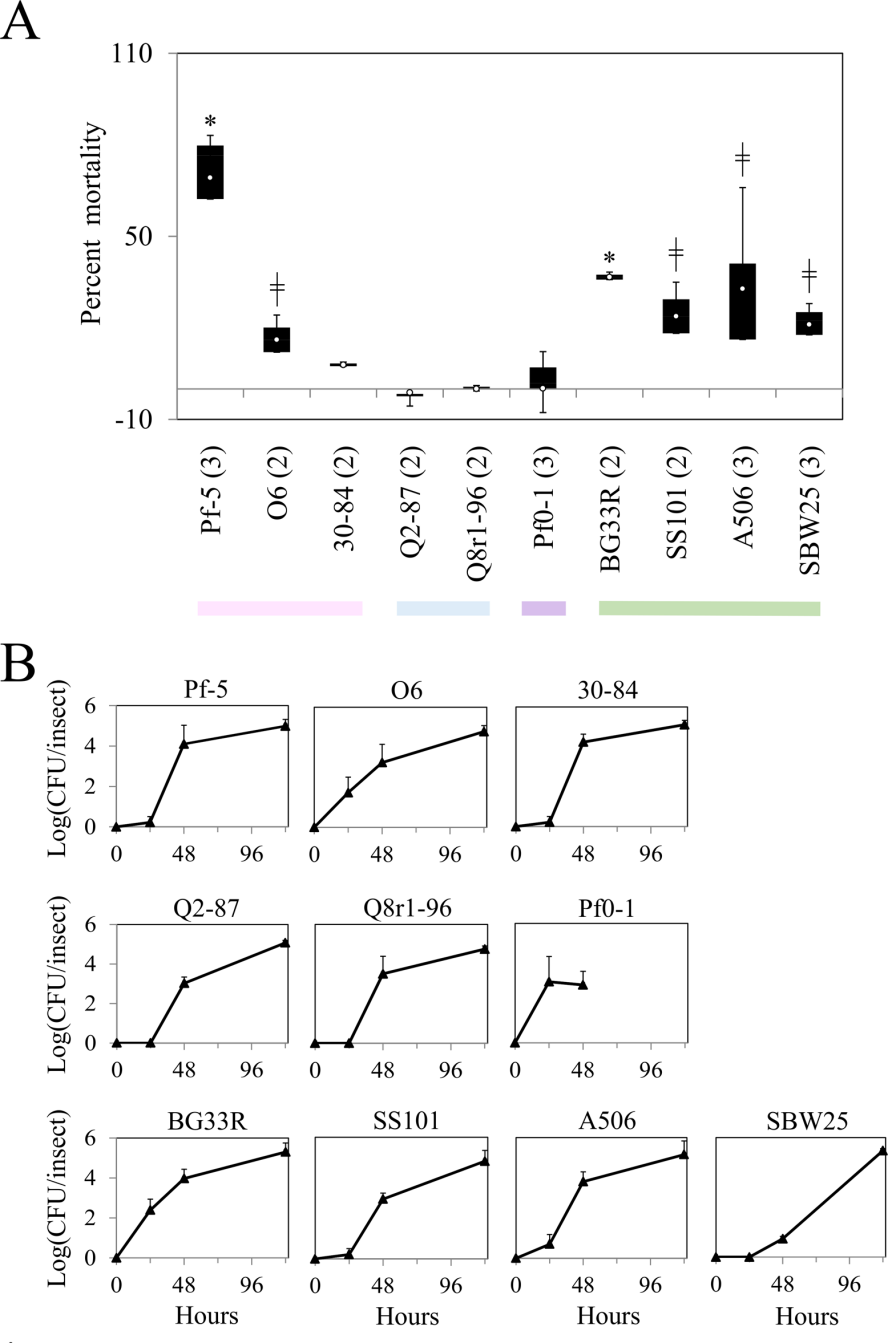
Oral toxicity of strains of *Pseudomonas* spp. to *D*. *melanogaster*. (A). The mortality of *D*. *melanogaster* was assessed 12 d after larvae were fed with yeast grains inoculated with ca. 7 log (CFU/plate). For each treatment, the percent mortality was calculated from counts of the number of adults per larvae in each replicate corrected for average larval to adult mortality in the control using the Schneider-Orelli formula. The white circle in each box shows the mean value from two or three experiments. The number of experiments evaluated for each strain is shown in parentheses. Boxes are bound at the top by the third quartile and at the bottom by the first quartile, with the whiskers representing the minimum and maximum values. An asterisk (*) denotes treatments that differed significantly from the control in all experiments and the double-cross symbol (╫) denotes treatments that differed significantly from the control in one experiment (P<0.05, d.f. = 2, χ^2^ test) (see S4 Table for data from individual experiments). The horizontal line indicates zero mortality. Colors denote the subgroup of the strain tested for insect toxicity: pink, *P*. *chlororaphis*; blue, *P*. *corrugata*; purple, *P*. *koreensis*; green, *P*. *fluorescens*. (B) The internal population size of each strain was estimated from surface-sterilized larvae at 24 h, 48 h, and 120 h. The population size of Pf0-1 was assessed only at 24 and 48 h. Bacterial population sizes were log transformed and the means of three replicate larvae are shown as triangular symbols. Error bars denoting standard errors are sometimes obscured by the symbols.

The ten strains of *Pseudomonas* spp. were evaluated for oral toxicity to *D*. *melanogaster* using a previously-developed non-invasive assay [10]. Averaging the results from three replicated experiments, 13% of the larvae died in the control treatment, in which larvae of *D*. *melanogaster* were fed with water-treated, killed yeast. In contrast, an average of 69% of the larvae fed *P*. *protegens* Pf-5-inoculated yeast died (Fig 5, S4 Table), which corresponds well with the previously-reported oral toxicity of Pf-5 to *D*. *melanogaster* [10]. The two other strains in the *P*. *chlororaphis* subgroup were evaluated in two experiments. Strain 30–84 caused no significant levels of mortality in either experiment whereas strain O6 caused a small but significant level of mortality in one experiment (Fig 5, S4 Table). In an earlier study, we reported that strain SBW25, a member of the *P*. *fluorescens* subgroup caused mortality of *D*. *melanogaster* [10]. In the present study, all four strains in the *P*. *fluorescens* subgroup caused significant mortality in at least one of the two to three experiments in which they were evaluated (Fig 5, S4 Table). Mortality caused by three of these strains (SS101, SBW25, and A506) varied among experiments, whereas BG33R caused significant mortality in both experiments in which it was evaluated. Neither strain in the *P*. *corrugata* subgroup (Q2-87 and Q8r1-96) nor Pf0-1 caused mortality of *D*. *melanogaster* in this assay (Fig 5, S4 Table). Populations of all ten strains increased inside larvae of *D*. *melanogaster* over the course of the experiment (Fig 5B), so it appears that all strains had the capacity to grow within the insect regardless of the magnitude of their influences on mortality.

**Fig 6:**
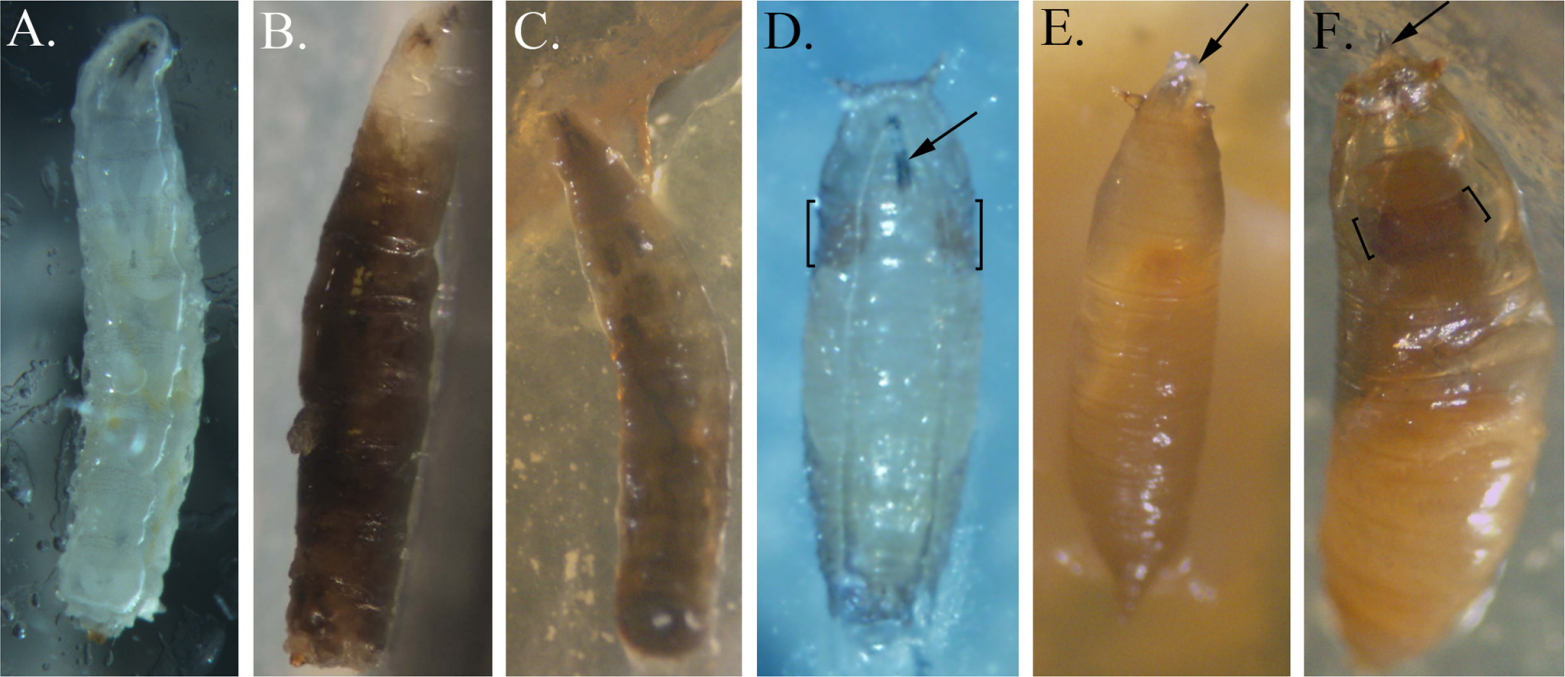
Larval and pupal phenotypes of *D*. *melanogaster* fed with strains of *Pseudomonas* spp. (A) Ventral view of a third instar control larva showing normal clear hemolymph and internal organs. (B) Side view of a dead SBW25-fed third instar larva showing systemic melanization of the hemolymph. (C) Dorsal view of a dead BG33R-fed third instar larva with complete melanization of the hemolymph. (D) Ventral view of a mid-stage control pupa showing normal extrusion of the mouthparts (arrow) and normal size of pupal eyes (brackets). (E) Dorsal view of a dead SS101-fed pre-pupa with extended mouthparts (arrow) and no head involution. (F) Dorsal view of a dead A506-fed pharate adult with extruded mouthparts caught within the pupal case (arrow). The head and eyes (brackets) are smaller and more recessed than in normal pupae.

When fed yeast inoculated with any of the four strains in the *P*. *fluorescens* subgroup, larvae consistently developed a systemic melanization of the hemolymph during third instar stage prior to larval death (Fig 6B & 6C compared to 6A), as described earlier for strain SBW25 [10]. The activation of the prophenoloxidase cascade leading to the production of melanin is an important component of *Drosophila* innate immunity that is normally tightly regulated to prevent damage to the host due to overproduction of quinones and excessive melanization [30,31,32]. This regulation appears to be relaxed when the insect ingests bacteria in the *P*. *fluorescens* subgroup. In addition, other larvae died as pupae because certain phases of normal pupariation did not occur or were less successful. In some cases, the animals failed to undergo head involution and their larval mouth hooks extended through the pupal case (Fig 6E compared to 6D). In some animals, head involution occurred but the mouth hooks were not extruded successfully leading to a failure to seal the anterior pupal case and subsequent death as pharate adults (Fig 6F). As these animals developed, the head and eyes were smaller than in pupae that had formed normally and most failed to emerge as adults (Fig 6F). Larvae that did not develop the systemic melanization appeared to pupate at normal rates, indicating that ingestion of strains in the *P*. *fluorescens* subgroup did not cause developmental delay as found with sub-lethal doses of Pf-5 (S7 Fig).

Ingestion of *P*. *protegens* Pf-5 is known to cause a delay in the development of *D*. *melanogaster* [10]. Accordingly, larvae that fed on Pf-5 pupated later than the control in this study (S7 Fig). Of the other nine strains evaluated in this study, none caused notable delays in metamorphosis (S7 Fig), which is consistent with an earlier report that strains Pf0-1 and SBW25 do not cause developmental delay [10]. Our results suggest that developmental delay of *D*. *melanogaster* is associated with ingestion of the most toxic strain, Pf-5, but is not a common outcome of ingesting bacteria in the *P*. *fluorescens* group.

## Discussion

The results of this study demonstrated that strains representing two lineages of the large and diverse *P*. *fluorescens* group exhibited toxicity against the lepidopteran *M*. *sexta* and the dipteran *D*. *melanogaster*. Three strains in the *P*. *chlororaphis* subgroup showed both injectable and oral toxicity to larvae of the tobacco hornworm, *M*. *sexta*, and one strain, *P*. *protegens* Pf-5, was also toxic to larvae of *D*. *melanogaster* when ingested. Three of four strains in the *P*. *fluorescens* subgroup were toxic when injected into *M*. *sexta*, and one strain consistently caused mortality of *D*. *melanogaster* when ingested. These results build upon a growing body of literature highlighting the insect toxicities of bacteria in the *P*. *fluorescens* group [12], including a recent study by Flury et al. [33] evaluating 26 strains of *Pseudomonas* spp. for toxicity to three lepidopteran species. Different insects and *Pseudomonas* strains were evaluated in the present study and in the study of Flury et al. [33] but the results of both studies show that strains in the *P*. *chlororaphis* subgroup and certain strains in the *P*. *fluorescens* subgroup exhibited insect toxicity whereas strains in the *P*. *corrugata* and *P*. *koreensis* subgroups did not exhibit consistent toxicity to insects. It remains to be seen if these taxonomic distinctions in insect toxicity hold up as more strains in the *corrugata* and *koreensis* subgroups are evaluated in the future.

Genes predicted to encode two distinct insect toxins (FitD/Mcf and Tc) known to be active in *Photorhabdus* spp. and other bacterial genera [34,35,36,37,38] are present in eight of the ten genomes of *Pseudomonas* spp. evaluated in this study. The three strains in the *P*. *chlororaphis* subgroup have the *fit* cluster, which is highly conserved among the strains in that subgroup that have been sequenced to date [15,16,33]. Seven of the ten strains have Tc gene clusters, identified from the presence of conserved domains and sequence similarity to regions encoding components of Tc toxins in *Photorhabdus* spp. and other entomopathogenic bacteria. Only *P*. *chlororaphis* 30–84 has both *fitD* and a Tc gene cluster, and bioinformatic analysis of the Tc gene cluster in strain 30–84 revealed characteristics of HGT. Here, we categorized the Tc gene clusters into six types based on their organization, phylogenies, and locations in the genome. For the most part, strains in a given subgroup shared Tc gene clusters of a single type, suggesting an ancestral inheritance of these clusters. Two of the ten strains evaluated in this study have two Tc clusters of different types in their genomes. Taken together, our analysis indicates that Tc gene clusters are inherited through a complex process involving HGT as well as vertical transmission through defined lineages of *Pseudomonas*.

A central objective of this study was to relate insect toxicity to the inventory of insect toxin genes in the genomes of diverse strains within the *P*. *fluorescens* group. The ten strains evaluated in this study have fully sequenced genomes that have been evaluated for the presence of genes for the production of exoenzymes, toxins, and metabolites that participate in the interactions of *Pseudomonas* spp. with insects, plants, and microorganisms [16]. Our results support a role for the FitD toxin in both injectable and oral toxicity to larvae of the lepidopteran *M*. *sexta*. All three strains (Pf-5, O6, and 30–84) that have a *fit* cluster caused injectable and oral toxicity to *M*. *sexta*, and a Δ*fitD* mutant of Pf-5 exhibited reduced oral toxicity (this study) and injectable toxicity to this insect [11,13]. These results support the known role of FitD in toxicity towards lepidopteran insects [11,13] and extend that role to oral toxicity against another insect pest, *M*. *sexta*. Only one of the seven strains lacking a *fit* cluster exhibited a significant level of oral toxicity to *M*. *sexta* in the single replicated experiment in which it was tested (S6 Fig). Therefore, *fitD* was the only factor identified in this study that contributed to oral toxicity of these ten strains against *M*. *sexta*. In contrast to oral toxicity, three strains lacking the *fit* cluster caused significant levels of injectable toxicity to *M*. *sexta*. These results provide convincing evidence that *fitD*, while important, is not the sole determinant of lepidopteran insect toxicity in the *P*. *fluorescens* group, as suggested earlier [11,13]. Identifying genes other than *fitD* that contribute to insect toxicity in these bacteria is a worthy goal for future study. Towards that end, this study identified strains within the *P*. *fluorescens* group that consistently cause injectable toxicity to *M*. *sexta* that could be explored in the future.

Tc gene clusters are among the many candidate genes that could contribute to injectable toxicity but we observed no correlation between the presence of Tc gene clusters and insect toxicity of the ten strains investigated here. We recognize, however, that different types of Tc gene clusters could vary in toxicity. For example, strains Q2-87 and Q8r1-96, which exhibited no insect toxicity in any of the three assays evaluated, have Type II and III Tc gene clusters whereas strains BG33R, A506 and SS101, which showed significant levels of injectable toxicity against *M*. *sexta*, have Type V Tc gene clusters. Strain BG33R, which also has a Type VI Tc gene cluster, consistently caused oral toxicity to *D*. *melanogaster*. The possibility that the Type V or VI Tc gene clusters contribute to insect toxicity is another worthy objective for future research. A *tccC* gene in *P*. *taiwanesis* is the only component of a Tc gene cluster that has been demonstrated to contribute to insect toxicity of *Pseudomonas* spp. [39]. Additional candidate insect toxicity genes may be identified from comparative genomic analysis [16] of strains A506, BG33R and SS101 versus strains that exhibited no toxicity to *M*. *sexta* in this study.

A different set of strains exhibited oral toxicity to the dipteran *D*. *melanogaster* versus the lepidopteran *M*. *sexta*, suggesting that distinct mechanisms of toxicity are operating in these insect hosts. Whereas *fitD* was the primary determinant of oral toxicity against *M*. *sexta*, as discussed above, this was not the case for toxicity against *D*. *melanogaster*. All three strains in the *P*. *chlororaphis* group have the *fit* cluster [16], but only Pf-5 consistently caused significant mortality of *D*. *melanogaster* in our assays. These results are consistent with those from a recent study demonstrating that FitD is not required for oral toxicity of *P*. *protegens* Pf-5 to *D*. *melanogaster* [40]. Instead, analogs of rhizoxin, orfamide A, and chitinase are the primary determinants of oral toxicity of Pf-5 against *D*. *melanogaster*. Rhizoxin is a 16-member macrolide that binds to β-tubulin, thereby interfering with microtubule dynamics during mitosis, and is toxic to many eukaryotes [41]. Orfamide A is a cyclic lipopeptide with surfactant properties that aids in bacterial motility across surfaces and solubilization of certain substrates [42], inhibition of some oomycetes and fungi [42,43,44], and toxicity to the green peach aphid [45]. Chitinases can degrade the peritrophic membrane, a chitin-based matrix in the insect mid-gut that functions in protection against mechanical and chemical damage and serves as a barrier to infection by pathogens [46]. Of the ten strains evaluated in this study, only Pf-5 produces orfamide A or rhizoxin analogs, but several strains that exhibited some level of toxicity to *D*. *melanogaster* have genes for the production of cyclic lipopeptides, chitinase, as well as other secondary metabolites that have not yet been evaluated for their potential roles in insect toxicity [16].

A striking observation of this study was the induction of melanization in adults that escaped mortality when fed any of the four strains in the *P*. *fluorescens* subgroup. Melanization is a conspicuous immune response that results in the production of quinones that are toxic to microorganisms [32]. It is intriguing that the strains in the *P*. *fluorescens* subgroup cause a pronounced melanization response in larvae and adults, but the animals that survive infection by *P*. *protegens* Pf-5, the most lethal strain of this study, do not exhibit this response. The mechanisms responsible for inducing this immune response are unknown but worthy of future study.

The results of this study highlight the specificity of the insect-bacterial interaction, as different strains caused mortality on the two insect hosts and when inoculated by feeding versus injection. Furthermore, evaluation of *fitD* mutants in this and a companion study [40] show that even a specific bacterial strain, such as *P*. *protegens* Pf-5, employs different lethality mechanisms on different insect hosts. Clearly, there are distinct mechanisms by which different strains of *Pseudomonas* spp. kill insects, and this study identified systems that could be explored to identify novel mechanisms of insect toxicity.

## Materials and Methods

### Generation of Multi-Locus Sequence Analysis (MLSA) phylogenetic tree

MAFFT v. 7.245 [47] was used to generate alignments for *gyrB*, *rpoD*, *rpoB*, and 16S rRNA from the type strains of the *P*. *fluorescens* group (S5 Table) and from the ten strains of *Pseudomonas* sp. listed in Table 1. Accession numbers for all sequences are provided in S5 Table. Alignments of gappy columns were trimmed using Gblocks [48]. The concatenated nucleotide sequences from each organism were used as input for a partitioned maximum-likelihood phylogenetic analysis using RAxML v. 8.1.21 [49], using GTR+GAMMA as the substitution model for each partition. Trees were generated according to the guidelines provided in the RAxML v. 8 user’s manual, with 100 individual maximum-likelihood searches performed and 450 bootstrap replicates were completed using the extended majority rule-based bootstrapping criterion [49].

**Table 1:** Strains of the *Pseudomonas fluorescens* group evaluated in this study.

Strain	Site where strain was isolated	Description	Source
***P*. *chlororaphis* subgroup:**			
*P*. *chlororaphis* 30–84	Wheat rhizosphere, Washington, USA	Suppresses take-all of wheat. Rif^R^, [50]	L.S. Pierson II, Texas A&M, College Station, TX USA
*P*. *chlororaphis* O6	Soil, Utah, USA	Suppresses several plant diseases [51]	A. Anderson, Utah State University, Logan Utah, USA
*P*. *protegens* Pf-5	Soil, Texas, USA	Suppresses seedling emergence diseases [24,52]. Also called JL4585.	C. Howell, USDA-ARS, College Station, TX, USA
*P*. *protegens* Pf-5 Δ*fitD*		Mutant of Pf-5 with a deletion in *fitD* [40]	
***P*. *corrugata* subgroup:**			
*P*. *brassicacearum* Q8r1-96	Wheat rhizosphere, Washington, USA	Suppresses take-all of wheat [53]	D. Weller, USDA-ARS, Pullman WA, USA
Q2-87	Wheat rhizosphere, Washington, USA	Suppresses take-all of wheat [54]	D. Weller, USDA-ARS, Pullman WA, USA
***P*. *koreensis* subgroup:**			
Pf0-1	Soil, Massachusetts, USA	Soil bacterium [55]	M. Silby, University of Massachusetts, Princeton, MA, USA
***P*. *fluorescens* subgroup:**			
SBW25	Sugar beet phyllosphere, Oxfordshire, UK	Phyllosphere bacterium [55]	G. Preston, Oxford University, UK
SBW25-Rif^R^		Spontaneous mutant of SBW25 selected for resistance to rifampicin, Rif^R^	J. M. Raaijmakers, Netherlands Institute of Ecology, Wageningen, The Netherlands
A506	Pear phyllosphere, California, USA	Suppresses fire blight of pear and apple; frost injury, fruit russet. Rif^R^ [7,56]	S. E. Lindow, University of California, Berkeley, CA, USA
SS101	Wheat rhizosphere, The Netherlands	Suppresses diseases caused by *Pythium* spp. and *Phytophthora* spp. Rif^R^ [57, 58]	J. M. Raaijmakers, Netherlands Institute of Ecology, Wageningen, The Netherlands
BG33R	Peach rhizosphere, South Carolina, USA	Suppresses the plant-parasitic nematode *Mesocriconema xenoplax* [59]	D. Kluepfel, USDA-ARS, Davis, CA, USA
BG33R-Rif^R^		Spontaneous mutant of BG33R selected for resistance to rifampicin, Rif^R.^	This study

Abbreviation: Rif^R^, resistant to rifampicin (100 μg/ml).

### Bioinformatic analysis of Tc clusters in genomes of *Pseudomonas spp.*

Using the nucleotide sequences of well-characterized Tc genes from *P*. *luminescens* W14 as queries, a BLASTN search was performed against the ten genomes of *P*. *fluorescens* to identify Tc genes. Conserved domains were identified by searching the Pfam database [60] and the conserved domain database of the National Center for Biotechnology Information (NCBI) [61]. Multiple sequence alignments of the Tc peptide sequences were executed using the MAFFT option in MegAlign Pro (DNAStar). Three unrooted phylogenetic trees were created using BIONJ with each putatively annotated amino acid sequence for each Tc component: A (TcaA, TcaB, and TcdA), B (TcaC and TcdB) and C (TccC). The G+C contents of *tcdA* and *tcdB* were determined using the MBCF Oligo Calculator (http://mbcf.dfci.harvard.edu/docs/oligocalc.html) and normalized to gene size. Significant differences in percent G+C from the genomic average were identified by chi-square analysis using a two-tailed *P*-value.

### Assessing injectable toxicity of *Pseudomonas* spp. to *M*. *sexta*

Injectable toxicities of ten strains of *Pseudomonas* spp. (Table 1) to *M*. *sexta* were assessed as described previously [11]. Briefly, bacteria were grown in 5 ml of King’s medium B (KMB) broth [62] for 24 h at 27°C. Cells were collected by centrifugation, washed, resuspended in sterile water at OD_600_ = 0.01 (ca. 7 log[CFU/ml]), and diluted to obtain cell densities specified in the Results. Dilutions of the cell suspensions were spread on KMB to determine titers of the inoculum injected into larvae of *M*. *sexta*. For each treatment, ten fifth instar larvae were injected between the second and third abdominal segments with 10 μl of water or bacterial suspension. Larvae were then placed in individual containers, maintained in an incubator at 16:8 h (L:D) and 27°C, and assessed for mortality over time. Each bacterial strain was evaluated in at least two replicated experiments. A chi-squared analysis was performed for each experiment, in which the negative control (sterile water) was compared to each treatment individually.

To monitor bacterial colonization of *M*. *sexta*, larvae were inoculated with ca. 5 log (CFU/larva) rifampicin-resistant mutants of *Pseudomonas* spp. and incubated as described above. Larvae with a healthy appearance were sampled immediately after inoculation and at 6, 12, 18, 24, and 30 h after injection. Each larva was surface-disinfested for 30 s in 70% ethanol, rinsed with sterile water, and then homogenized for 30 s in 10 ml of sterile distilled H_2_O using a Tekmar SDT Tissumizer (Cincinnati, OH, USA). Serial dilutions were prepared from the resulting homogenate and plated onto KMB containing rifampicin (100 μg/ml) and cycloheximide (50 μg/ml) to select for the injected strains. Plates were incubated for 24 h at 27°C prior to enumeration of bacterial colonies. The experiment was done twice with similar results and a representative experiment is presented.

### Assessing oral toxicity of *Pseudomonas* spp. to *M*. *sexta*

Oral toxicity of *Pseudomonas* spp. to *M*. *sexta* was assessed on excised leaves of tomato (cv. Patio). Excised leaves were dipped in an aqueous bacterial suspension prepared from overnight cultures grown in KMB broth; cells were collected by centrifugation and suspended in sterile water to an OD_600_ of 0.5, which corresponds to approximately 9 log (CFU/ml). Leaves were placed in plastic containers lined with moistened paper towels. Containers were placed in a lighted growth chamber at 27°C to allow epiphytic bacterial populations to develop on leaf surfaces. After the 24 h incubation period, three leaflets were removed from each treatment, placed in separate tubes containing 10 mM potassium phosphate buffer (pH 7.0), and vortexed. The number of CFU of bacterial strains was assessed by serial dilution of leaflet washings on KMB. For each treatment, fifteen larvae, ranging in size from 18 to 30 mg, were placed on leaves in separate containers (i.e., one larvae per container; 15 containers/treatment). Containers were placed in a growth chamber maintained at 27°C with 17:7 h (L:D), 40–50% relative humidity). Larval mortality was assessed at 2, 4, and 6 d after their placement on leaves. One to five of the fifteen larvae feeding on water-treated leaves died in each of the seven experiments, which was probably due in part to injuries caused by handling the larvae as they were placed on the leaves. Due to logistical constraints, only four treatments could be assessed in each experiment. A chi-squared analysis was performed to compare each bacterial treatment to the water treatment within each experiment.

### Assessing oral toxicity of *Pseudomonas* spp. to *D*. *melanogaster*

Oral toxicity of *Pseudomonas* spp. to *D*. *melanogaster* was assessed using a non-invasive assay described previously [10]. Briefly, adult flies were transferred to Petri plates containing Apple Agar (http://cshprotocols.cshlp.org/content/2011/9/pdb.recO65672.short) prepared without Nipagin and supplemented by placing killed yeast grains (20 mg) on the solidified agar surface. Plates were incubated for four hours at 25°C to allow egg lay. From these plates, thirty eggs were transferred aseptically to the surface of non-nutritive agar (2% wt/vol agar in water) with 2 to 3 mg killed yeast grains distributed on the agar surface in a 35mm Petri plate. On Day 1, the number of first instar larvae was determined by counting the number of empty egg cases. On Day 2, 200 μl of a yeast suspension was added to the middle of the plate to serve as a food source for second instar larvae. The yeast suspension was prepared by dissolving 0.2 g yeast in 1.2 ml of sterile water (control) or a bacterial suspension (7.4 ± 0.6 log[CFU/ml]), prepared as described above. The plates were then incubated at 22°C and, starting at Day 4, larvae were fed with 100 μl of a yeast suspension (0.2 mg yeast/1.2 ml sterile water) at 48-h intervals as long as live larvae were observed in the dish. Numbers of pupae and adults were counted daily. Each bacterial strain was tested in at least two experiments having three replications per strain (S4 Table). For each experiment, percent mortality for each treatment was calculated from the number of adults on Day 12 per the number of larvae on Day 1 and averaged over the replicate plates. Mean percent mortality values for each experiment were corrected for the mortality in the control yeast-only plates using the Schneider-Orelli formula (% corrected adult mortality = [(% adult mortality of treated larvae—% adult mortality of control larvae)/(100—% adult mortality of control larvae)] [63]. A chi-squared analysis of the corrected mortality data was used to detect significant differences of treatments from the control in each experiment.

Population sizes of the bacterial strains internal to larvae or pupae of *D*. *melanogaster* were estimated from surface-sterilized larvae. Three larvae were placed in a drop of a freshly-made sterilizing solution (2.5 ml bleach and 45 μl 0.01% Triton-X in 10 ml water) for 60 seconds and then transferred serially through four drops of sterile water and placed individually into a 1.5 ml microcentrifuge tube. Each insect was then homogenized in 50 μl of sterile distilled water, serially diluted, and the dilutions were spread on KMB. Plates were incubated overnight at 27°C prior to counting colonies that exhibited the green-blue fluorescence characteristic of the inoculated strains.

Larval and pupal morphologies were documented through images captured by a digital camera mounted on a dissecting scope with images adjusted only for contrast and brightness in Photoshop (Adobe Systems Inc., San Jose, CA, USA) as needed.

## Supporting Information

S1 Fig Unrooted distance-based phylogeny of toxin complex (Tc) A components. The A-component tree contains seven TcaA sequences, seven TcaB sequences, and one TcdA sequence from the ten strains within the *P*. *fluorescens* group examined in this study (shown in red font) as well as BLASTP hits with greater than 75% query coverage and 50% identity to one or more of these sequences. The tree also includes all of the characterized A-component peptide sequences (TcaA, TcbA, TcdA) from *P*. *luminescens* W14 (shown in blue font). Phylogenetic relationships support the placement of the Tc clusters into the six types (Types I to VI), with components of the same Tc type from different strains grouping within the same clades. Boxes show the Tc type and are colored to denote the subgroup of strains shown in red font: pink, *chlororophis*; blue, *corrugata*; green, *fluorescens*, as depicted in Fig 2.
(PDF)

S2 Fig Unrooted distance-based phylogeny of toxin complex (Tc) B components. The B component tree contains eight TcaC sequences and one TcdB sequence from the ten strains within the *P*. *fluorescens* group examined in this study (shown in red font) as well as BLASTP hits with greater than 75% query coverage and 50% identity to one or more of these sequences. The tree also includes all of the characterized B-component peptide sequences (TcaC and TcdB) from *P*. *luminescens* W14 (shown in blue font). All TcaC sequences grouped together with homologs from other *Pseudomonas* sp. in a single large clade, whereas the Type I TcdC sequence fell outside of this clade and grouped with the only BLASTP hit outside of the *Pseudomonas* genus. Phylogenetic relationships support the placement of the Tc clusters into the six types (Types I to VI), with components of the same Tc type from different strains grouping together. Boxes show the Tc type and are colored to denote the subgroup of strains shown in red font: pink, *chlororophis*; blue, *corrugata*; purple, *koreensis*; green, *fluorescens*, as depicted in Fig 2.
(PDF)

S3 Fig Unrooted distance-based phylogeny of toxin complex (Tc) C components. The C-component tree contains 15 TccC sequences from the ten strains within the *P*. *fluorescens* group examined in this study (shown in red font) as well as BLASTP hits with greater than 75% query coverage and 50% identity to one or more of these sequences. The tree also includes all characterized C-component peptide sequences (TccC) from *P*. *luminescens* W14 (shown in blue font). The six types of Tc clusters (I to VI) fall into distinct clades, but both the Type II and Type III TccC sequences are dispersed in the tree. Boxes show the Tc type and are colored to denote the subgroup of strains shown in red font: pink, *chlororaphis*; blue, *corrugata*; purple, *koreensis*; green, *fluorescens*, as depicted in Fig 2.
(PDF)

S4 FigLethality of strains in the *P*. *fluorescens* group to the tobacco hornworm, *M*. *sexta*, post-injection.Lethality of strains in the *P*. *fluorescens* group to fifth instar of the tobacco hornworm, *M*. *sexta*. Mortality was assessed at 24h (■), 48h (■), or 72h (■) following injection with ca. 5 log(CFU per larva) or water, as a control. Three experiments (A, B and C), each evaluating ten replicate larvae per treatment, are presented. The 72 h observations for experiment A are shown in Fig 3. Asterisks represent a significant difference in mortality between a treatment and the control (*P*<0.05, d.f. = 1, χ2 test).(TIF)

S5 FigLethality of strains in the *P*. *fluorescens* group to the tobacco hornworm, *M*. *sexta*, post-injection.Lethality of high cell densities of strains in the *P*. *fluorescens* group to fifth instar of the tobacco hornworm, *M*. *sexta*. Mortality was assessed at 24h (■), 48h (■), or 72h (■) following injection with ca. 6 log(CFU per larva) or water, as a control. Two experiments (A and B), each evaluating ten replicate larvae per treatment, are presented. Asterisks represent a significant difference in mortality between a treatment and the control (*P*<0.05, d.f. = 1, χ2 test).(TIF)

S6 FigOral toxicity of strains in the *P*. *fluorescens* group to the tobacco hornworm, *M*. *sexta*.Cumulative mortality of *M*. *sexta* was assessed by counting the number of dead larvae at 2 d (■), 4 d (■) and 6 d (■) after larvae were placed on tomato leaves supporting epiphytic populations of the specified bacterial strain. Strain Pf0-1 *gacA*+ (also called LK194) is a derivative of strain Pf0-1 with a chromosomal insertion of *gacA* [64]. Controls were larvae on leaves that were not inoculated with bacteria. (A-E) Each panel shows the results from an individual experiment, with fifteen replicate larvae evaluated per treatment in each experiment. Values that differ significantly from the control at the designated time are shown with an asterisk (*P*<0.05) or a diamond (*P*<0.10) (d.f. = 1, χ2 test). The epiphytic population size of each strain on tomato leaflets, determined at the time that larvae were placed on the leaves, is shown below each graph.(TIF)

S7 Fig **Developmental time course of *D*. *melanogaster* after ingestion of strains in the A) *P*. *chlororaphis*, B) *P*. *koreensis* or *P*. *corrugata*, or C) *P*. *fluorescens* subgroups.** Second instar larvae were fed with a yeast suspension having no bacteria (black) or amended with bacterial strains. Initial population sizes of bacterial strains [log (CFU/plate)] are shown to the right of each panel. The percentage of larvae that pupated, counted as prepupae and/or pupae were determined over time. The percentage of larvae that emerged as adults is shown at the 288 hpi time point. Values represent the mean and standard errors from three replicates per treatment, with each replicate evaluating the larvae and adults that developed from 30 eggs. A Δ*gacA* mutant of Pf-5 (JL4975) [65] was included as a negative control, as it was shown previously to lack toxicity to *D*. *melanogaster* [10].
(TIF)

S1 Table All *tcaA/tcdA*-like gene products have the VRP1 (PF03538) domain. (DOC)

S2 Table All *tcaC/tcdB*-like gene products have SpvB (PF03534) and MidN/MidC (PF12255) domains. (DOC)

S3 Table All *tccC*-like gene products have RhsA (COG3209) and Rhs-core (TIGR03696) domains. (DOC)

S4 Table Summary of experiments evaluating mortality of *D*. *melanogaster*. (DOC)

S5 Table Accession numbers for housekeeping genes of type strains in the *Pseudomonas fluorescens* group used in phylogenetic analysis. (DOC)
