# Supplementary figures and images for: Characterization of Toxin Complex Gene Clusters and Insect Toxicity of Bacteria Representing Four Subgroups of Pseudomonas fluorescens
Source: PLoS One. 2016 Aug 31;11(8):e0161120. doi: 10.1371/journal.pone.0161120 (PMC5006985; doi:10.1371/journal.pone.0161120)

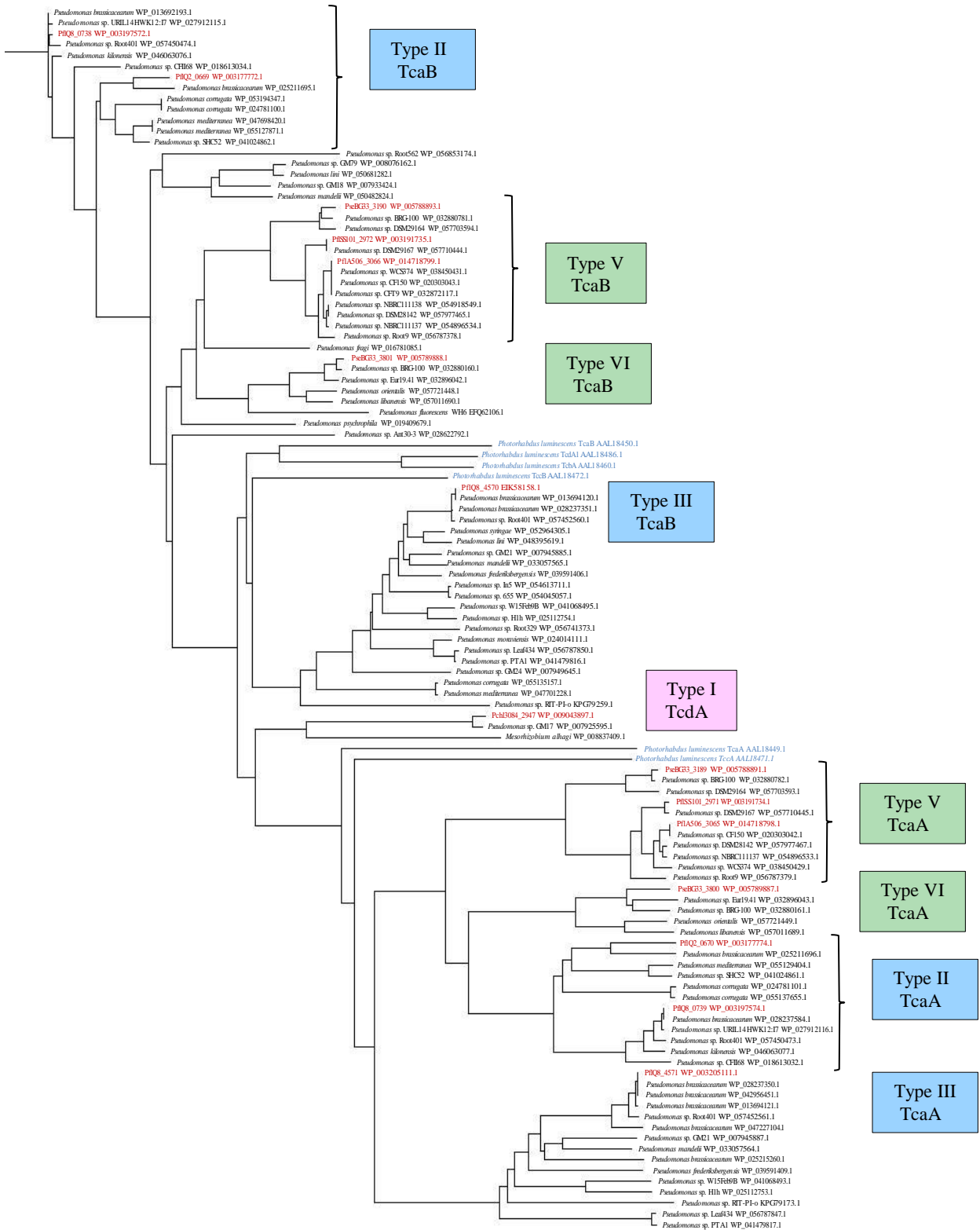

Supplement: S1 Fig — The A-component tree contains seven TcaA sequences, seven TcaB sequences, and one TcdA sequence from the ten strains within the P. fluorescens group examined in this study (shown in red font) as well as BLASTP hits with greater than 75% query coverage and 50% identity to one or more of these sequences. The tree also includes all of the characterized A-component peptide sequences (TcaA, TcbA, TcdA) from P. luminescens W14 (shown in blue font). Phylogenetic relationships support the placement of the Tc clusters into the six types (Types I to VI), with components of the same Tc type from different strains grouping within the same clades. Boxes show the Tc type and are colored to denote the subgroup of strains shown in red font: pink, chlororophis; blue, corrugata; green, fluorescens, as depicted in Fig 2. (PDF) [file pone.0161120.s001.pdf]

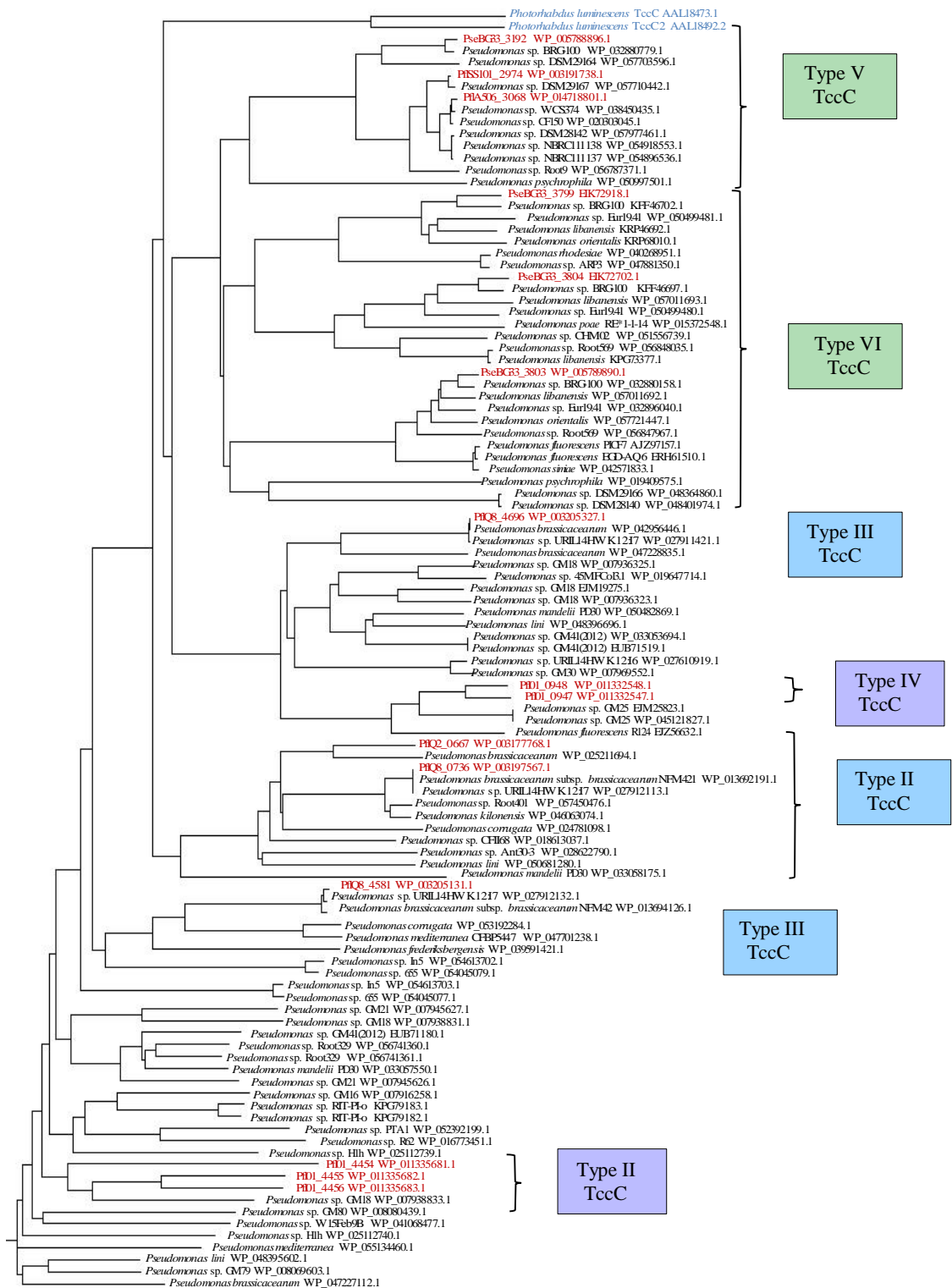

Supplement: S3 Fig — The C-component tree contains 15 TccC sequences from the ten strains within the P. fluorescens group examined in this study (shown in red font) as well as BLASTP hits with greater than 75% query coverage and 50% identity to one or more of these sequences. The tree also includes all characterized C-component peptide sequences (TccC) from P. luminescens W14 (shown in blue font). The six types of Tc clusters (I to VI) fall into distinct clades, but both the Type II and Type III TccC sequences are dispersed in the tree. Boxes show the Tc type and are colored to denote the subgroup of strains shown in red font: pink, chlororaphis; blue, corrugata; purple, koreensis; green, fluorescens, as depicted in Fig 2. (PDF) [file pone.0161120.s003.pdf]

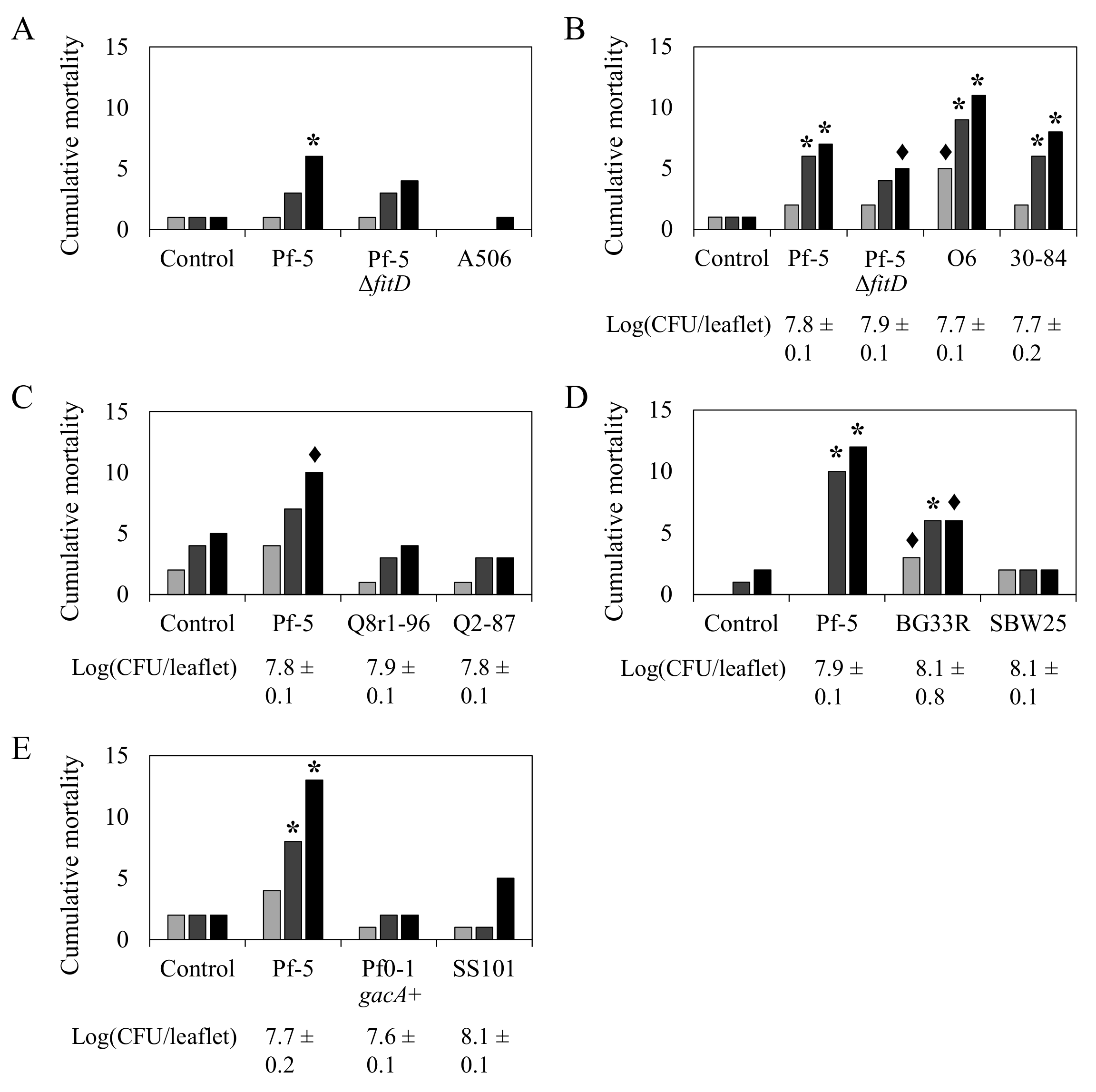

Supplement: S6 Fig — Cumulative mortality of M. sexta was assessed by counting the number of dead larvae at 2 d (■), 4 d (■) and 6 d (■) after larvae were placed on tomato leaves supporting epiphytic populations of the specified bacterial strain. Strain Pf0-1 gacA+ (also called LK194) is a derivative of strain Pf0-1 with a chromosomal insertion of gacA [64]. Controls were larvae on leaves that were not inoculated with bacteria. (A-E) Each panel shows the results from an individual experiment, with fifteen replicate larvae evaluated per treatment in each experiment. Values that differ significantly from the control at the designated time are shown with an asterisk (P<0.05) or a diamond (P<0.10) (d.f. = 1, χ2 test). The epiphytic population size of each strain on tomato leaflets, determined at the time that larvae were placed on the leaves, is shown below each graph. (TIF) [file pone.0161120.s006.tif]

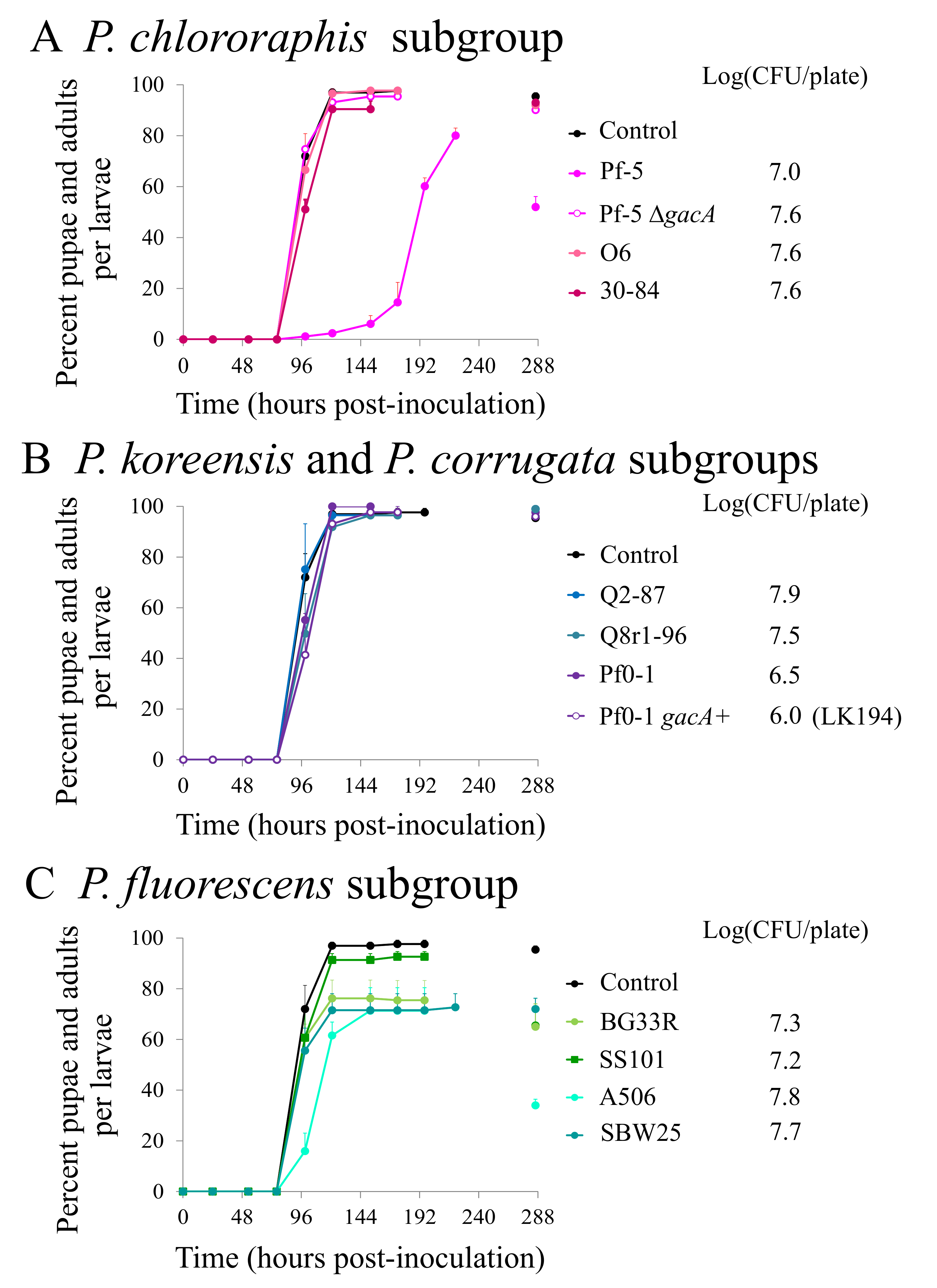

Supplement: S7 Fig — Developmental time course of D. melanogaster after ingestion of strains in the A) P. chlororaphis, B) P. koreensis or P. corrugata, or C) P. fluorescens subgroups. Second instar larvae were fed with a yeast suspension having no bacteria (black) or amended with bacterial strains. Initial population sizes of bacterial strains [log (CFU/plate)] are shown to the right of each panel. The percentage of larvae that pupated, counted as prepupae and/or pupae were determined over time. The percentage of larvae that emerged as adults is shown at the 288 hpi time point. Values represent the mean and standard errors from three replicates per treatment, with each replicate evaluating the larvae and adults that developed from 30 eggs. A ΔgacA mutant of Pf-5 (JL4975) [65] was included as a negative control, as it was shown previously to lack toxicity to D. melanogaster [10]. (TIF) [file pone.0161120.s007.tif]
