# Supplementary material for: Characterization of Toxin Complex Gene Clusters and Insect Toxicity of Bacteria Representing Four Subgroups of Pseudomonas fluorescens
Source: PLoS One. 2016 Aug 31;11(8):e0161120. doi: 10.1371/journal.pone.0161120 (PMC5006985; doi:10.1371/journal.pone.0161120)
Supplement: S1 Table — (DOC) [file pone.0161120.s008.doc]

**S1 Table. All *tcaA/tcdA*-like gene products possess**

the VRP1 (PF03538) domain

| **Locus tag** | **AA** | **Alignment^a^** | **E-value^b^** |
| --- | --- | --- | --- |
| Pchl3084_2947 | 2729 | 57-292 | 9.70E-12 |
| PflQ8_4571 | 1203 | 34-311 | 8.70E-27 |
| PflQ8_0739 | 844 | 29-318 | 2.20E-45 |
| PflQ2_0670 | 843 | 30-319 | 8.20E-48 |
| PseBG33_3800 | 931 | 5-282 | 7.30E-45 |
| PseBG33_3189 | 869 | 5-219 | 1.70E-33 |
| PflA506_3065 | 878 | 5-229 | 3.10E-35 |
| PflSS101_2971 | 878 | 6-229 | 1.60E-35 |

^a/b^ alignment and e-values taken from pfam.sanger.ac.uk
