# Supplementary material for: Characterization of Toxin Complex Gene Clusters and Insect Toxicity of Bacteria Representing Four Subgroups of Pseudomonas fluorescens
Source: PLoS One. 2016 Aug 31;11(8):e0161120. doi: 10.1371/journal.pone.0161120 (PMC5006985; doi:10.1371/journal.pone.0161120)
Supplement: S2 Table — (DOC) [file pone.0161120.s009.doc]

**S2 Table. All *tcaC/tcdB***-like gene products possess SpvB (PF03534) and MidN/MidC (PF12255) domains

|  |  | **SpvB** | | **MidN** | | **MidC** | |
| --- | --- | --- | --- | --- | --- | --- | --- |
| **Locus tag** | **AA** | **Alignment^a^** | **E-value^b^** | **Alignment** | **E-value** | **Alignment** | **E-value** |
| Pchl3084_2950 | 2459 | 42-316 | 9.90E-78 | 647-824 | 6.90E-41 | 369-879 | 5.90E-32 |
| Pfl01_4453 | 1488 | 27-310 | 1.80E-102 | 628-806 | 6.80E-56 | 855-997 | 2.00E-41 |
| PflQ8_0737 | 1493 | 28-302 | 7.70E-101 | 627-806 | 5.80E-50 | 854-1000 | 1.00E-41 |
| PflQ8_4580 | 1447 | 27-303 | 1.30E-91 | 627-809 | 2.60E-49 | 857-1000 | 1.60E-30 |
| PflQ2_0668 | 1495 | 28-303 | 1.50E-99 | 627-805 | 1.60E-50 | 853-1002 | 1.50E-40 |
| PseBG33_3802 | 1527 | 29-316 | 1.90E-103 | 642-821 | 2.80E-48 | 876-1025 | 5.90E-36 |
| PseBG33_3191 | 1477 | 30-314 | 2.50E-103 | 644-814 | 1.40E-44 | 861-1009 | 3.40E-39 |
| PflA506_3067 | 1476 | 30-314 | 8.60E-99 | 643-815 | 4.20E-45 | 862-1010 | 4.70E-39 |
| PflSS101_2973 | 1476 | 30-314 | 5.09E-100 | 643-815 | 3.10E-44 | 862-1010 | 1.40E-39 |

^a/b^ alignment and e-values taken from pfam.sanger.ac.uk
