# Supplementary material for: Characterization of Toxin Complex Gene Clusters and Insect Toxicity of Bacteria Representing Four Subgroups of Pseudomonas fluorescens
Source: PLoS One. 2016 Aug 31;11(8):e0161120. doi: 10.1371/journal.pone.0161120 (PMC5006985; doi:10.1371/journal.pone.0161120)
Supplement: S3 Table — (DOC) [file pone.0161120.s010.doc]

**S3 Table. All *tccC*-like gene products possess RhsA (COG3209) and Rhs-core (TIGR03696**) domains

|  |  | **RhsA** | | **Rhs core** | |
| --- | --- | --- | --- | --- | --- |
| **Locus tag** | **AA** | **Alignment^a^** | **E-value^b^** | **Alignment** | **E-value** |
| Pfl01_0947 | 893 | 177-657 | 3.91E-29 | 574-648 | 5.86E-25 |
| Pfl01_0948 | 942 | 12-675 | 9.09E-30 | 574-647 | 3.04E-21 |
| Pfl01_4456 | 891 | 26-616 | 6.03E-23 | 544-623 | 1.4E-24 |
| Pfl01_4455 | 944 | 41-625 | 4.69E-18 | 544-623 | 7.3E-26 |
| Pfl01_4454 | 928 | 38-623 | 2.49E-29 | 543-622 | 2.71E-24 |
| PflQ8_4696 | 905 | 70-657 | 4.13E-24 | 577-657 | 6.93E-16 |
| PflQ8_0736 | 927 | 23-648 | 1.71E-29 | 572-652 | 9.5E-30 |
| PflQ2_0667 | 929 | 23-649 | 8.08E-34 | 569-649 | 1.67E-30 |
| PseBG33_3804 | 916 | 6-663 | 6.43E-25 | 581-660 | 2.04E-28 |
| PseBG33_3803 | 995 | 68-669 | 4.11E-28 | 587-667 | 9.95E-30 |
| PseBG33_3799 | 698 | 144-670 | 5.17E-24 | 583-663 | 4.14E-27 |
| PseBG33_3192 | 966 | 89-667 | 7.22E-29 | 586-666 | 1.29E-32 |
| PflA506_3068 | 964 | 1-668 | 2.00E-32 | 586-666 | 1.33E-27 |
| PflSS101_2974 | 967 | 1-668 | 9.80E-33 | 586-666 | 8.42E-30 |

^a/b^ alignment and e-values taken from CDD: NCBI's conserved domain database
