# Supplementary material for: Characterization of Toxin Complex Gene Clusters and Insect Toxicity of Bacteria Representing Four Subgroups of Pseudomonas fluorescens
Source: PLoS One. 2016 Aug 31;11(8):e0161120. doi: 10.1371/journal.pone.0161120 (PMC5006985; doi:10.1371/journal.pone.0161120)
Supplement: S4 Table — (DOC) [file pone.0161120.s011.doc]

***S4 Table. Summary of experiments evaluating mortality of Drosophila melanogaster.***

| **Treatment** | **Strain #** | **Experiment R4** | | | | **Experiment R8** | | | | **Experiment R9** | | |
| --- | --- | --- | --- | --- | --- | --- | --- | --- | --- | --- | --- | --- |
|  |  | % egg hatch^a^ | Log (CFU/plate)^b^ | % Mortality^c^ | % Mortality (Corrected)^d^ | % egg hatch^a^ | Log (CFU/plate) ^b^ | % Mortality^c^ | % Mortality (Corrected)^d^ | % egg hatch^a^ | % Mortality^c^ | % Mortality (Corrected)^d^ |
| Control |  | 91 ± 2 | 0.0 | 5 ± 1 |  | 89 ± 6 | 0.0 | 16 ± 1 |  | 92 ± 3 | 17 ± 5 |  |
| Pf-5 | JL4585 | 95 ± 2 | 8.2 | 53 ± 4 | 48 ± 4 | 92 ± 5 | 7.0 | 92 ± 4 | 77 ± 4 | 94 ± 3 | 100 | 83 ± 0 |
| 30-84 | JL4867 | 94 ± 2 | 8.3 | 12 ± 3 | 8 ± 3 | 89 ± 5 | 6.8 | 25 ± 3 | 9 ± 3 |  |  |  |
| O6 | JL4871 | 93 ± 4 | 8.3 | 13 ± 3 | 7 ± 3 | 89 ± 5 | 6.8 | 40 ± 3 | 24 ± 3 |  |  |  |
| Pf0-1 | JL4803, LK077 | 97 | 8.0 | -2 | 3 | 90 ± 4 | 5.7 | 10 ± 5 | -6 ± 5 | 93 ± 4 | 25 ± 10 | 8 ± 10 |
| Pf0-1 *gacA*+ | LK194 |  |  |  |  | 92 ± 5 | 5.2 | 13 ± 7 | -3 ± 7 | 93 ± 4 | 47 ± 9 | 30 ± 9 |
| Q2-87 | JL4988 | 93 ± 2 | 8.1 | 5 ± 3 | 0 ± 3 | 89 ± 1 | 7.1 | 14 ± 3 | -2 ± 3 |  |  |  |
| Q8r1-96 | JL4816 | 93 ± 1 | 8.1 | 6 ± 3 | 1 ± 3 | 88 ± 1 | 6.7 | 15 ± 2 | -1 ± 2 |  |  |  |
| SBW25 | JL4802 | 95 ± 3 | 8.0 | 33 ± 4 | 28 ± 4 | 89 ± 6 | 6.9 | 29 ± 6 | 13 ± 6 | 94 ± 3 | 39 ± 12 | 22 ± 12 |
| BG33R | JL4870 | 94 ± 3 | 8.1 | 40 ± 9 | 35 ± 9 | 90 ± 2 | 6.5 | 54 ± 3 | 38 ± 3 |  |  |  |
| A506 | LK001 | 96 ± 2 | 8.2 | 71 ± 2 | 66 ± 2 | 90 ± 2 | 7.0 | 32 ± 2 | 16 ± 2 | 94 ± 3 | 33 ± 3 | 16 ± 3 |
| SS101 | LK002 | 93 ± 3 | 8.2 | 40 ± 7 | 35 ± 7 | 89 ± 3 | 6.4 | 29 ± 6 | 13 ± 6 |  |  |  |

^a^ For each replicate, 30 eggs of *D. melanogaster* were placed on a Petri plate containing a non-nutritive agar and yeast grains as a food source. One day later, the number of first instar larvae was determined by counting the number of empty egg cases on each plate and dividing by 30 to calculate % egg hatch. Values are the mean of three replicate plates, except for in experiment R4, where the control had five replicates and Pf0-1 had one plate only. Mean values are followed by the standard error of the mean.

^b^ Two days after eggs were placed on the plates, a yeast suspension was added to the agar surface. For the control treatment, yeast grains were suspended in sterile water before they were placed on the plate. For other treatments, yeast grains were in a suspension of bacterial cells from strains shown in the columns at the left. The population size of each bacterial strain on the agar surface (Log[CFU/plate]) was estimated by dilution plating as described in the Materials and Methods and S7 Fig.

^c^ % Mortality is the inverse of % survival, which was calculated from the number of adults (counted at day 12) divided by the number of first instar larvae (counted at day 1) in each replicate. Values are the mean of three replicate plates, except for in experiment R4, where the control had five replicates and Pf0-1 had one plate only. Mean values are followed by the standard error of the mean.

^d^ The percent mortality per treatment group was corrected for average larval to adult mortality in the control using the Schneider-Orelli formula: % mortality (corrected) = [(% mortality of treated larvae - % mortality of control larvae)/(100 - % mortality of control larvae)] (Puntener 1981). The corrected mortality for each replicate plate was then averaged. Red font denotes values that differ significantly from the control based on chi-squared analysis of each treatment plate. Values are the mean of three replicate plates, except for in experiment R4, where the control had five replicates and Pf0-1 had one plate only. Mean values are followed by the standard error of the mean.
