# Supplementary material for: Characterization of Toxin Complex Gene Clusters and Insect Toxicity of Bacteria Representing Four Subgroups of Pseudomonas fluorescens
Source: PLoS One. 2016 Aug 31;11(8):e0161120. doi: 10.1371/journal.pone.0161120 (PMC5006985; doi:10.1371/journal.pone.0161120)
Supplement: S5 Table — (DOC) [file pone.0161120.s012.doc]

**S5 Table. Accession numbers for housekeeping genes of type strains in the *Pseudomonas fluorescens* group used in phylogenetic analysis.**

| **Type Strain** | ***gyrB*** | ***rpoB*** | ***rpoD*** | **16S** |
| --- | --- | --- | --- | --- |
| *Pseudomonas aeruginosa* | AB039386 | AJ717442 | AB039607 | Z76672 |
| *Pseudomonas agarici* | AB039457 | AJ717477 | AB039563 | Z76652 |
| *Pseudomonas antarctica* | FN554169 | FN554727 | FN554450 | AJ537601 |
| *Pseudomonas asplenii* | AB039455 | AJ717432 | AB039593 | AB021397 |
| *Pseudomonas azotoformans* | AB039411 | AJ717458 | AB039547 | D84009 |
| *Pseudomonas brassicacearum* | AM084675 | AJ717436 | AM084334 | AF100321 |
| *Pseudomonas brenneri* | FN554176 | AJ717482 | FN554457 | AF268968 |
| *Pseudomonas cedrina* | FN554178 | AJ717424 | FN554459 | AF064461 |
| *Pseudomonas chlororaphis subsp. aurantiaca* | FN554171 | AJ717421 | FN554452 | DQ682655 |
| *Pseudomonas chlororaphis subsp. aureofaciens* | FN554172 | AJ717426 | FN554453 | AY509898 |
| *Pseudomonas chlororaphis subsp. chlororaphis* | D86019 | AJ717478 | D86036 | Z76673 |
| *Pseudomonas corrugata* | AB039460 | AJ717487 | AB039566 | D84012 |
| *Pseudomonas costantinii* | FN554180 | FN554732 | FN554461 | AF374472 |
| *Pseudomonas extremorientalis* | FN554182 | FN554733 | FN554464 | AF405328 |
| *Pseudomonas fluorescens* | D86016 | AJ717451 | D86033 | D84013 |
| *Pseudomonas fragi* | FN554184 | AJ717444 | FN554466 | AF094733 |
| *Pseudomonas frederiksbergensis* | AM084676 | AJ717465 | AM084335 | FR750403 |
| *Pseudomonas fuscovaginae* | FN554185 | AJ717433 | FN554467 | FJ483519 |
| *Pseudomonas gessardii* | FN554186 | AJ717438 | FN554468 | AF074384 |
| *Pseudomonas grimontii* | FN554188 | AJ717439 | FN554470 | AF268029 |
| *Pseudomonas jessenii* | AM293562 | AJ717447 | FN554473 | AF068259 |
| *Pseudomonas kilonensis* | AM084677 | AJ717472 | AM084336 | AJ292426 |
| *Pseudomonas koreensis* | AM293563 | FN554737 | FN554476 | AF468452 |
| *Pseudomonas libanensis* | FN554195 | AJ717454 | FN554477 | AF057645 |
| *Pseudomonas lini* | FN554196 | AJ717466 | FN554478 | AY035996 |
| *Pseudomonas lurida* | JN589908 | HE586402 | HE586451 | AJ581999 |
| *Pseudomonas lundensis* | FN554197 | AJ717428 | FN554479 | AB021395 |
| *Pseudomonas mandelii* | FN554200 | AJ717435 | FN554482 | AF058286 |
| *Pseudomonas marginalis* | AB039448 | AJ717425 | AB039575 | Z76663 |
| *Pseudomonas mediterranea* | AM084678 | AJ717449 | AM084337 | AF386080 |
| *Pseudomonas meridiana* | FN554203 | FN554740 | FN554485 | AJ537602 |
| *Pseudomonas migulae* | FN554204 | AJ717446 | FN554486 | AF074383 |
| *Pseudomonas mohnii* | AM293561 | FN554741 | FN554487 | AM293567 |
| *Pseudomonas moorei* | AM293560 | FN554742 | FN554489 | AM293566 |
| *Pseudomonas moraviensis* | FN554206 | FN554743 | FN554490 | AY970952 |
| *Pseudomonas mucidolens* | AB039409 | AJ717427 | AB039546 | AB681967 |
| *Pseudomonas orientalis* | FN554209 | AJ717434 | FN554493 | AF064457 |
| *Pseudomonas palleroniana* | FN554213 | FN554747 | FN554497 | AY091527 |
| *Pseudomonas panacis* | FN554214 | FN554748 | FN554498 | AY787208 |
| *Pseudomonas poae* | FN554219 | FN554751 | FN554504 | AJ492829 |
| *Pseudomonas proteolytica* | FN554220 | FN554752 | FN554505 | AJ537603 |
| *Pseudomonas psychrophila* | FN554221 | AJ717464 | FN554506 | AB041885 |
| *Pseudomonas reinekei* | AM293559 | FN554754 | FN554508 | AM293565 |
| *Pseudomonas rhodesiae* | FN554225 | AJ717431 | FN554511 | AF064459 |
| *Pseudomonas salomonii* | FN554226 | FN554756 | FN554512 | AY091528 |
| *Pseudomonas simiae* | FN554227 | FN554757 | FN554513 | AJ936933 |
| *Pseudomonas synxantha* | AB039415 | AJ717420 | AB039550 | AB680171 |
| *Pseudomonas taetrolens* | AB039412 | AJ717423 | AB039523 | D84027 |
| *Pseudomonas thivervalensis* | AM084679 | AM084680 | AM084338 | AF100323 |
| *Pseudomonas tolaasii* | AB039423 | AJ717467 | AB039561 | AF255336 |
| *Pseudomonas trivialis* | FN554230 | FN554762 | FN554515 | AJ492831 |
| *Pseudomonas umsongensis* | AM293564 | FN554763 | FN554516 | AF468450 |
| *Pseudomonas vancouverensis* | FN554232 | AJ717473 | FN554517 | AJ011507 |
| *Pseudomonas veronii* | FN554233 | AJ717445 | FN554518 | AF064460 |
