# Supplementary material for: Characterization of Toxin Complex Gene Clusters and Insect Toxicity of Bacteria Representing Four Subgroups of Pseudomonas fluorescens
Source: PLoS One. 2016 Aug 31;11(8):e0161120. doi: 10.1371/journal.pone.0161120 (PMC5006985; doi:10.1371/journal.pone.0161120)
Supplement: S2 Fig — The B component tree contains eight TcaC sequences and one TcdB sequence from the ten strains within the P. fluorescens group examined in this study (shown in red font) as well as BLASTP hits with greater than 75% query coverage and 50% identity to one or more of these sequences. The tree also includes all of the characterized B-component peptide sequences (TcaC and TcdB) from P. luminescens W14 (shown in blue font). All TcaC sequences grouped together with homologs from other Pseudomonas sp. in a single large clade, whereas the Type I TcdC sequence fell outside of this clade and grouped with the only BLASTP hit outside of the Pseudomonas genus. Phylogenetic relationships support the placement of the Tc clusters into the six types (Types I to VI), with components of the same Tc type from different strains grouping together. Boxes show the Tc type and are colored to denote the subgroup of strains shown in red font: pink, chlororophis; blue, corrugata; purple, koreensis; green, fluorescens, as depicted in Fig 2. (PDF) [file pone.0161120.s002.pdf]

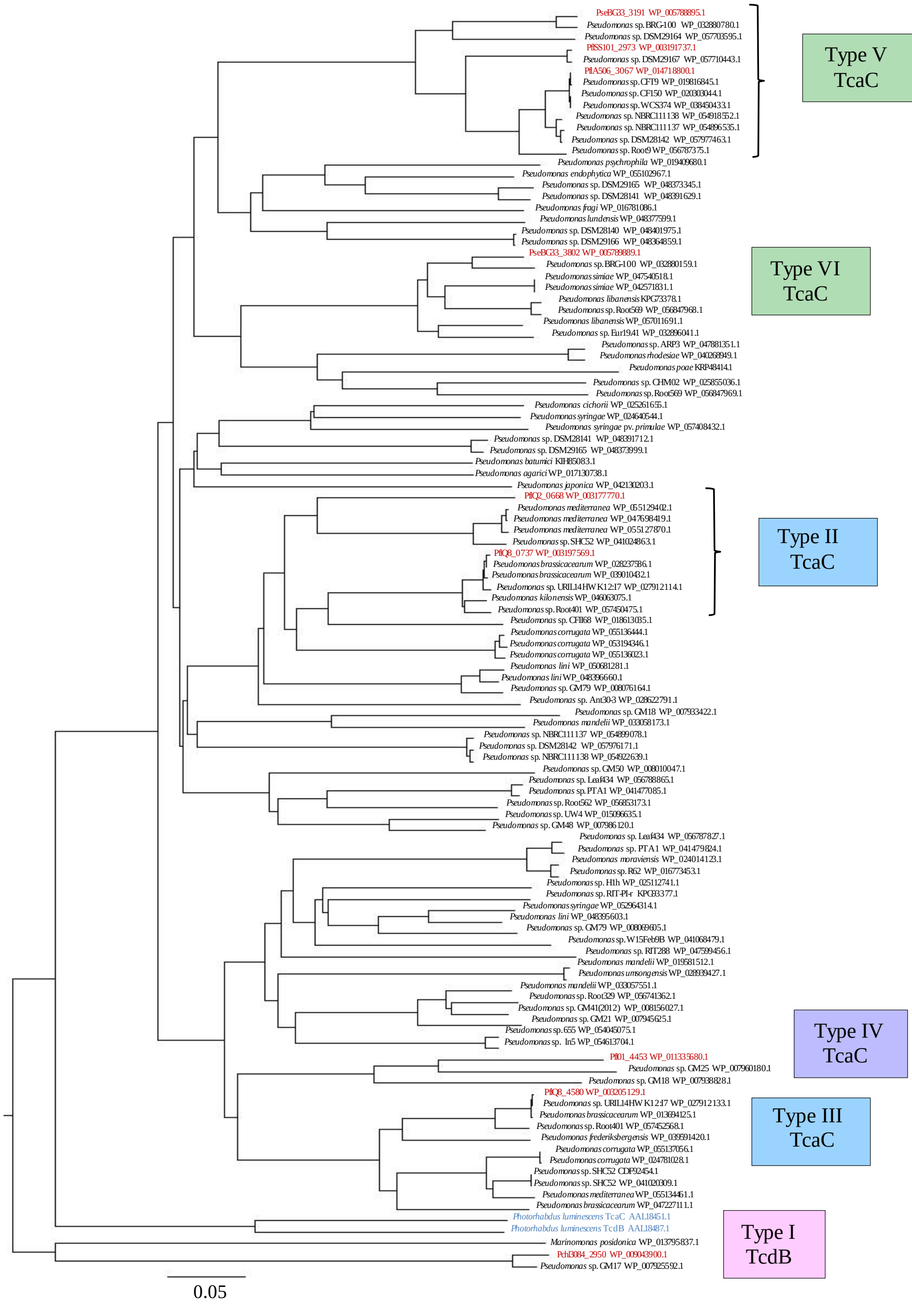

PseBG33_3191 WP_005788895.1
Pseudomonas sp. BRG-100 WP_032880780.1
Pseudomonas sp. DSM29164 WP_057703595.1
PflSS101_2973 WP_003191737.1
Pseudomonas sp. DSM29167 WP_057710443.1
PflA506_3067 WP_014718800.1
Pseudomonas sp. CFT9 WP_019816845.1
Pseudomonas sp. CF150 WP_020303044.1
Pseudomonas sp. WCS374 WP_038450433.1
Pseudomonas sp. NBRC111138 WP_054918552.1
Pseudomonas sp. NBRC111137 WP_054896535.1
Pseudomonas sp. DSM28142 WP_057977463.1
Pseudomonas sp. Root9 WP_056787375.1
Type V TcaC
Pseudomonas psychrophila WP_019409680.1
Pseudomonas endophytica WP_055102967.1
Pseudomonas sp. DSM29165 WP_048373345.1
Pseudomonas sp. DSM28141 WP_048391629.1
Pseudomonas fragi WP_016781086.1
Pseudomonas lundensis WP_048377599.1
Pseudomonas sp. DSM28140 WP_048401975.1
Pseudomonas sp. DSM29166 WP_048364859.1
PseBG33_3802 WP_005789889.1
Pseudomonas sp. BRG-100 WP_032880159.1
Pseudomonas simiae WP_047540518.1
Pseudomonas simiae WP_042571831.1
Pseudomonas libanensis KPG73378.1
Pseudomonas sp. Root569 WP_056847968.1
Pseudomonas libanensis WP_057011691.1
Pseudomonas sp. Eur1 9.41 WP_032896041.1
Type VI TcaC
Pseudomonas sp. ARP3 WP_047881351.1
Pseudomonas rhodesiae WP_040268949.1
Pseudomonas poae KRP48414.1
Pseudomonas sp. CHM02 WP_025855036.1
Pseudomonas sp. Root569 WP_056847969.1
Pseudomonas cichorii WP_025261655.1
Pseudomonas syringae WP_024640544.1
Pseudomonas syringae pv. primulae WP_057408432.1
Pseudomonas sp. DSM28141 WP_048391712.1
Pseudomonas sp. DSM29165 WP_048373999.1
Pseudomonas batumici KIH85083.1
Pseudomonas agarici WP_017130738.1
Pseudomonas japonica WP_042130203.1
PflQ2_0668 WP_003177770.1
Pseudomonas mediterranea WP_055129402.1
Pseudomonas mediterranea WP_047698419.1
Pseudomonas mediterranea WP_055127870.1
Pseudomonas sp. SHC52 WP_041024863.1
PflQ8_0737 WP_003197569.1
Pseudomonas brassicacearum WP_028237586.1
Pseudomonas brassicacearum WP_039010432.1
Pseudomonas sp. URIL14HWK12:I7 WP_027912114.1
Pseudomonas kilonensis WP_046063075.1
Pseudomonas sp. Root401 WP_057450475.1
Type II TcaC
Pseudomonas sp. CFII68 WP_018613035.1
Pseudomonas corrugata WP_055136444.1
Pseudomonas corrugata WP_053194346.1
Pseudomonas corrugata WP_055136023.1
Pseudomonas lini WP_050681281.1
Pseudomonas lini WP_048396660.1
Pseudomonas sp. GM79 WP_008076164.1
Pseudomonas sp. Ant30-3 WP_028622791.1
Pseudomonas sp. GM18 WP_007933422.1
Pseudomonas mandelii WP_033058173.1
Pseudomonas sp. NBRC111137 WP_054899078.1
Pseudomonas sp. DSM28142 WP_057976171.1
Pseudomonas sp. NBRC111138 WP_054922639.1
Pseudomonas sp. GM50 WP_008010047.1
Pseudomonas sp. Leaf434 WP_056788865.1
Pseudomonas sp. PTA1 WP_041477085.1
Pseudomonas sp. Root562 WP_056853173.1
Pseudomonas sp. UW4 WP_015096635.1
Pseudomonas sp. GM48 WP_007986120.1
Pseudomonas sp. Leaf434 WP_056787827.1
Pseudomonas sp. PTA1 WP_041479824.1
Pseudomonas moraviensis WP_024014123.1
Pseudomonas sp. R62 WP_016773453.1
Pseudomonas sp. H1h WP_025112741.1
Pseudomonas sp. RIT-PI-r KPG93377.1
Pseudomonas syringae WP_052964314.1
Pseudomonas lini WP_048395603.1
Pseudomonas sp. GM79 WP_008069605.1
Pseudomonas sp. W15Feb9B WP_041068479.1
Pseudomonas sp. RIT288 WP_047599456.1
Pseudomonas mandelii WP_019581512.1
Pseudomonas umsongensis WP_028939427.1
Pseudomonas mandelii WP_033057551.1
Pseudomonas sp. Root329 WP_056741362.1
Pseudomonas sp. GM41(2012) WP_008156027.1
Pseudomonas sp. GM21 WP_007945625.1
Pseudomonas sp. 655 WP_054045075.1
Pseudomonas sp. In5 WP_054613704.1
Type IV TcaC
Pfl01_4453 WP_011335680.1
Pseudomonas sp. GM25 WP_007960180.1
Pseudomonas sp. GM18 WP_007938828.1
PflQ8_4580 WP_003205129.1
Pseudomonas sp. URIL14HWK12:I7 WP_027912133.1
Pseudomonas brassicacearum WP_013694125.1
Pseudomonas sp. Root401 WP_057452568.1
Pseudomonas frederiksbergensis WP_039591420.1
Pseudomonas corrugata WP_055137056.1
Pseudomonas corrugata WP_024781028.1
Pseudomonas sp. SHC52 CDF92454.1
Pseudomonas sp. SHC52 WP_041020309.1
Pseudomonas mediterranea WP_055134461.1
Pseudomonas brassicacearum WP_047227111.1
Type III TcaC
Photorhabdus luminescens TcaC AAL18451.1
Photorhabdus luminescens TcdB AAL18487.1
Marinomonas posidonica WP_013795837.1
PchI3084_2950 WP_009043900.1
Pseudomonas sp. GM17 WP_007925592.1
Type I TcdB
0.05
